# Passive immunotherapy for N-truncated tau ameliorates the cognitive deficits in two mouse Alzheimer’s disease models

**DOI:** 10.1093/braincomms/fcaa039

**Published:** 2020-04-06

**Authors:** Veronica Corsetti, Antonella Borreca, Valentina Latina, Giacomo Giacovazzo, Annabella Pignataro, Paraskevi Krashia, Francesca Natale, Sara Cocco, Marco Rinaudo, Francesca Malerba, Rita Florio, Roberta Ciarapica, Roberto Coccurello, Marcello D’Amelio, Martine Ammassari-Teule, Claudio Grassi, Pietro Calissano, Giuseppina Amadoro

**Affiliations:** f1 European Brain Research Institute (EBRI), 00161 Rome, Italy; f2 Humanitas University Laboratory of Pharmacology and Brain Pathology, Neuro Center, 20089 Milan, Italy; f3 Institute of Neuroscience, 20129 Milan, Italy; f4 IRCSS Santa Lucia Foundation, 00143 Rome, Italy; f5 Department of Medicine, University Campus Bio-Medico, 00128 Rome, Italy; f6 Department of Science and Technology for Humans and Environment, University Campus Bio-medico, 00128 Rome, Italy; f7 Fondazione Policlinico Universitario A. Gemelli IRCCS, 00168 Rome, Italy; f8 Institute for Complex Systems (ISC), CNR, 00185 Rome, Italy; f9 Institute of Human Physiology, Università Cattolica del Sacro Cuore, 00168 Rome, Italy; f10 Institute of Translational Pharmacology (IFT)–National Research Council (CNR), 00133 Rome, Italy

**Keywords:** tau protein, tauopathies, Alzheimer’s disease, tau cleavage, immunotherapy

## Abstract

Clinical and neuropathological studies have shown that tau pathology better correlates with the severity of dementia than amyloid plaque burden, making tau an attractive target for the cure of Alzheimer’s disease. We have explored whether passive immunization with the 12A12 monoclonal antibody (26–36aa of tau protein) could improve the Alzheimer’s disease phenotype of two well-established mouse models, Tg2576 and 3xTg mice. 12A12 is a cleavage-specific monoclonal antibody which selectively binds the pathologically relevant neurotoxic NH_2_26-230 fragment (i.e. NH_2_htau) of tau protein without cross-reacting with its full-length physiological form(s). We found out that intravenous administration of 12A12 monoclonal antibody into symptomatic (6 months old) animals: (i) reaches the hippocampus in its biologically active (antigen-binding competent) form and successfully neutralizes its target; (ii) reduces both pathological tau and amyloid precursor protein/amyloidβ metabolisms involved in early disease-associated synaptic deterioration; (iii) improves episodic-like type of learning/memory skills in hippocampal-based novel object recognition and object place recognition behavioural tasks; (iv) restores the specific up-regulation of the activity-regulated cytoskeleton-associated protein involved in consolidation of experience-dependent synaptic plasticity; (v) relieves the loss of dendritic spine connectivity in pyramidal hippocampal CA1 neurons; (vi) rescues the Alzheimer’s disease-related electrophysiological deficits in hippocampal long-term potentiation at the CA3-CA1 synapses; and (vii) mitigates the neuroinflammatory response (reactive gliosis). These findings indicate that the 20–22 kDa NH_2_-terminal tau fragment is crucial target for Alzheimer’s disease therapy and prospect immunotherapy with 12A12 monoclonal antibody as safe (normal tau-preserving), beneficial approach in contrasting the early Amyloidβ-dependent and independent neuropathological and cognitive alterations in affected subjects.

## Teaser

Intercepting the pathologically relevant species without interfering with the physiological form(s) of protein is one of the challenges of tau-directed immunotherapy. The present study shows that antibody-mediated selective neutralization of the toxic N-truncated tau fragment in hippocampi from two transgenic Alzheimer’s disease mice significantly improves the synaptic functions.

## Graphical Abstract

**Graphical Abstract:**
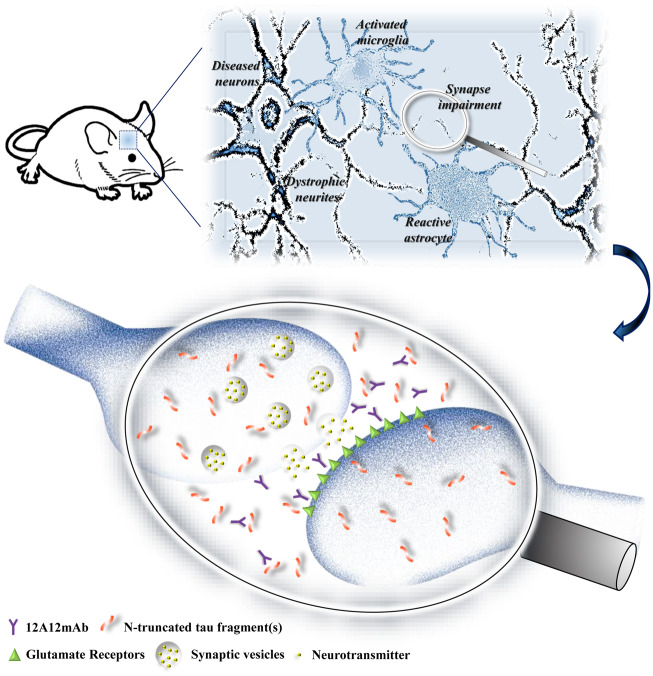

## Introduction

Recent *in vitro* and *in vivo* data have highlighted a crucial role of proteolytic fragments of tau protein, in particular those derived from truncation at its N-terminal domain, in the initiation/progression of Alzheimer’s disease and other related tauopathies, thus paving the way for their potential use as therapeutic targets or as biomarkers for diagnosing dementia and/or monitoring disease progression (Avila *et al.*, 2016*a*; Quinn *et al.*, 2018; Sebastián-Serrano *et al.*, 2018). On the one hand, tau cleavage may generate amyloidogenic fragments that initiate its aggregation which, in turn, can cause toxicity (Wang *et al.*, 2010). On the other hand, tau proteolysis may result in production of noxious, both intracellular and extracellular, truncated species which drive neurodegeneration independently of aggregative pathway(s) and in a fragment-dependent manner as a result of their deleterious action on pre- and/or post-synaptic functions and/or their secretion and transcellular propagation (Quinn *et al.*, 2018).

Extracellular cleaved tau is toxic to neurons by increasing the Aβ production (Bright *et al.*, 2015) and/or by impairing synaptic plasticity (Fà *et al.*, 2016; Florenzano *et al.*, 2017; Borreca *et al.*, 2018; Hu *et al.*, 2018). Hyperphosphorylation and caspase-3 cleavage of tau (Asp421), which promote aggregation, also favour the protein secretion *in vitro* (Plouffe *et al.*, 2012). The amino-terminal projection domain of human tau—which interacts with the plasma membrane (Brandt *et al.*, 1995) and undergoes early conformational changes in human tauopathies including Alzheimer’s disease (Combs *et al.*, 2016, 2017)—is endowed with deleterious action(s), mainly at nerve endings (King *et al.*, 2006; Ittner *et al.*, 2010; Amadoro *et al.*, 2012; Zhou *et al.*, 2017). The N-terminus extremity of tau, despite the lack of the microtubule binding domains which abnormally aggregate to form paired helical filaments, is prone to come into higher order of oligomerization (Feinstein *et al.*, 2016) and is specifically released into the extracellular space in an *in situ* tauopathy model (Kim *et al.*, 2010), suggesting a potential role for molecular ‘templating’ in the propagation of neurofibrillary lesions. Soluble C-terminally truncated tau species are also preferentially secreted from synaptosomes of Alzheimer’s disease brains (Sokolow *et al.*, 2015) and in conditioned media from patient-derived induced pluripotent stem cell cortical neurons (Bright *et al.*, 2015; Kanmert *et al.*, 2015; Sato *et al.*, 2018). Interestingly, although full-length tau is found in CSF from healthy humans, a heterogeneous population of fragments—including the NH_2_-terminal and/or prolin-rich domain—is mainly discernible in Alzheimer’s disease patients (Johnson *et al.*, 1997; Portelius *et al.*, 2008; Meredith *et al.*, 2013; Amadoro *et al.*, 2014; Chen *et al.*, 2019; Cicognola *et al.*, 2019). Exosomes-associated NH_2_-derived tau fragments are also detected in CSF from Alzheimer’s disease patients (Saman *et al.*, 2012) and a different CSF pattern of NH_2_-derived tau fragments may reflect disease-specific neurodegenerative processes (Borroni *et al.*, 2009). Consistently, passive immunotherapy with antibody targeting the N-terminal projection domain of full-length human tau has shown to be beneficial in Alzheimer’s disease transgenic (Tg) mice by improving the cognitive deficits (Yanamandra *et al.*, 2013; Dai *et al.*, 2015; Subramanian *et al.*, 2017) and blocking the seeding/spreading of tau pathology (Dai *et al.*, 2018). Both intracerebroventricular infusion and peripheral administration of anti-tau antibodies specific for N-terminal 25–30 epitopes are curative in P301S mice model of tauopathy, by preventing the brain atrophy and ameliorating the motor/sensorimotor functions (Yanamandra *et al.*, 2013, 2015). Immunization with antibody directed against the N-terminal end of full-length tau protein (Dai *et al.*, 2017) significantly reduced the level of amyloid precursor protein (APP), amyloid-β peptides (Aβ40) and Aβ42 in CA1 region of Alzheimer’s disease animal models, indicating that tau-based immunotherapy is actually able to restore the Aβ-dependent and/or independent synaptic dysfunction(s) which occur at early stages in Alzheimer’s disease and other related dementias (Pedersen and Sigurdsson, 2015; Panza *et al.*, 2016). However, albeit tau appears to be the main factor underlying the development and progression of Alzheimer’s disease (Kametani and Hasegawa *et al.*, 2018; Castellani and Perry, 2019), its expression at physiological level is required for neuronal functions underlying learning and memory (Pooler *et al.*, 2014; Regan *et al.*, 2017) and its down-regulation, even if moderate, has been proved to have deleterious effects, both *in vitro* and *in vivo* (Biundo *et al.*, 2018; Velazquez *et al.*, 2018). As a consequence, the development of selectively targeting antibodies against pathogenic tau may have a unique therapeutic advantage by leading to valuable, beneficial effects—in the absence of unwanted consequences due to the ‘loss of function’ of normal protein—in the cure of human, chronic neurodegenerative tauopathies which are sometimes expected to require long-term treatments with multiple and high-dose administrations of drugs (Kontsekova *et al.*, 2014; Elmaleh *et al.*, 2019).

In this framework, we developed a neo-epitope antibody directed against the N-terminal sequence of human tau protein DRKD(_25_)-QGGYTMHQDQE (Amadoro *et al.*, 2012) which encompasses a conserved cleavage-site sequence previously identified in cellular and animal Alzheimer’s disease models (Corsetti *et al.*, 2008) and in human Alzheimer’s disease brains (Rohn *et al.*, 2002). 12A12 (formerly Caspase-Cleaved protein-NH_2_4268 tau antiserum, Amadoro *et al.*, 2012) is a monoclonal antibody (mAb) which recognizes the newly created Δ-_25_NH_2_tau(Q26–36aa)-terminus of degradation product(s) of tau without cross-reacting with the same amino acidic stretch from full-length isoforms of intact, normal protein (Amadoro *et al.*, 2019; Supplementary Fig. 1). The pathologically relevant NH_2_tau 26–44aa stretch, which is the minimal active moiety of a neurotoxic 20–22 kDa NH_2_-derived tau peptide (aka NH_2_htau), accumulates at Alzheimer’s disease pre-synaptic terminals (Amadoro *et al.*, 2006, 2010, 2012; Corsetti *et al.*, 2015) and is present in CSFs from living patients suffering from Alzheimer’s disease and other non-Alzheimer’s disease neurodegenerative diseases (Amadoro *et al.*, 2014). Interestingly, this peptide is able to negatively impact on normal synaptic function(s) *in vitro* (Florenzano *et al.*, 2017) and *in vivo* (Borreca *et al.*, 2018), suggesting that its antibody-mediated selective clearance can have important clinical and translational implications in contrasting the earliest neuropathological and cognitive alterations associated with human tauopathies, including Alzheimer’s disease (Bright *et al.*, 2015; Sokolow *et al.*, 2015; Barthélemy *et al.*, 2016*a*, *b*; Sato *et al.*, 2018; Cicognola *et al.*, 2019).

In this study, we explored the potentially beneficial immunotherapeutic power of the 12A12mAb by means of its intravenous (i.v.) administration in two lines of Alzheimer’s disease Tg animals with different genetic backgrounds, such as Tg2576 carrying the APP KM670/671NL Swedish mutation and 3xTg mice expressing the amyloid precursor protein KM670/671NL Swedish mutation, tauP301L, PS1M146V human transgenes. Relevantly, unlike other murine or humanized NH_2_tau-directed immunotherapeutic antibodies (Yanamandra *et al.*, 2013, 2015; Dai *et al.*, 2015, 2017, 2018; Subramanian *et al.*, 2017; Qureshi *et al.*, 2018), 12A12mAb reacts with the 20–22 kDa neurotoxic NH_2_-truncated tau but not with the physiological full-length form of protein (Corsetti *et al.*, 2008; Amadoro *et al.*, 2012) advocating its *in vivo* use as safe, more harmless and personalized medicine treatment to slow progressing human tauopathies.

## Materials and methods

### Animals

All experiments involving animals were performed in accordance with the ARRIVE guidelines and were carried out in accordance with the ethical guidelines of the European Council Directive (2010/63/EU); experimental approval was obtained from the Italian Ministry of Health (protocol # 524/2017 PR; 554/2016-PR). Only male subjects were used to avoid changes in female hormone state that can affect cognitive data. All efforts were made to minimize the number of animals used and suffering.

One, 3- and 6-month-old Tg2576 and 3xTg mice (Tg-Alzheimer’s disease) (*n* = 8–10 per group/treatment) and age-matched wild-type (WT) controls (*n* = 8–10 per group/treatment) were used in this study.

Heterozygous Tg2576 mice overexpressing the APP695 with the Swedish mutation (APP KM670/671NL, TgHuAPP695swe: Tg2576) in a hybrid genetic background (87% C57BL/6 × 12.5% SJL) (Hsiao *et al.*, 1996) were subsequently backcrossed to C57BL/6xSJL F1 females and the offspring were genotyped to confirm the presence of human mutant APP DNA sequence by PCR. WT littermates were used as controls.

The homozygous 3xTg mice harbouring human amyloid precursor protein KM670/671NL Swedish mutation and tauP301L transgenes with knock-in PS1M146V under the control of the mouse Thy1.2 promoter were obtained from The Jackson Laboratory (https://www.jax.org/strain/004807). Mice were bred on the mixed C7BL/6; 129X1/SvJ; 129S1/Sv genetic background and genotypes were confirmed by PCR on tail biopsies (Oddo *et al.*, 2003). B6129SF2/J strain mice, used as WT controls in the present study, were the offspring of a cross between C57BL/6J females (B6) and 129S1/SvImJ males (129S); they are commonly used as controls for genetically engineered strains generated with 129-derived embryonic stem cells and maintained on a mixed B6; 129 background (https://www.jax.org/strain/101045). The housing conditions (four or five animals per cage) in pathogen-free facilities were controlled (temperature 22°C, 12 h light/12 h dark cycles, humidity 50–60%) with *ad libitum* access to chow and water.

### Immunization scheme

The N-terminal tau 12A12 antibody (26–36aa) was produced and characterized by monoclonal antibodies core facility at EMBL—Monterotondo, Rome, Italy (Dott. Alan Sawyer), as previously described in Florenzano *et al.* (2017). 12A12mAb was purified from hybridoma supernatants according to standard procedures and its purity was determined using sodium dodecyl sulphate-polyacrylamide gel electrophoresis and Coomassie staining. In detail, the hybridoma supernatant was precipitated by ammonium sulphate (336 g/l). After precipitation, the solution was centrifuged at 10 000 g for 1 h and the pellet was dissolved in phosphate-buffered-saline (PBS) and dialyzed against the same buffer. The solution was centrifuged at 10 000 g for 30 min and loaded on a HiTrap Protein G HP (GE Healthcare) equilibrated with PBS. The column was washed with PBS (5 column volumes). 121A12mAb was eluted with 0.1 M Glycine-HCl, pH 2.7. The fractions containing the antibody were neutralized by 1 M Tris-HCl, pH 9.0, collected and immediately dialyzed against PBS. 121A12mAb concentration was determined by measuring the absorbance at 280 nm. The average yield was 8 mg/l of cell supernatant. 12A12mAb was ≥95% pure and contained ≤1 U/mg of endotoxin (LAL Chromogenic Endotoxin quantitation kit; Thermo Scientific).

To minimize experimental variability, all mice were initially grouped according to their body weight and age and mice from the same litter were finally assigned to different groups. For each animal strain (Tg2576, 3xTg), the grouped mice were randomized into: (i) WT mice treated with saline vehicle; (ii) WT mice treated with 12A12mAb (30 μg/dose); (iii) age-matched Tg-Alzheimer’s disease mice treated with saline or non-specific mouse Immunoglobulin (IgG) (normal mouse IgG, Santa Cruz sc-2025, 30 μg/dose); and (iv) age-matched Tg-Alzheimer’s disease mice treated with 12A12mAb (30 μg/dose) or non-specific mouse IgG (normal mouse IgG, Santa Cruz sc-2025, 30 μg/dose). Animals were infused over 14 days with two weekly injections administered on two alternate days to the lateral vein of the tail. The dose and route of immunization were based on prior studies using Alzheimer’s disease Tg mice (Castillo-Carranza *et al.*, 2015). In details, mice were placed in a restrainer (Braintree Scientific), and an inch of the tail was shaved and placed in warm water to dilate veins. After injection via the lateral tail vein, mice were returned to home cages and kept under general observation. Abnormalities in overall health, home-cage nesting, sleeping, feeding, grooming, body weight and condition of the fur of animals were noted.

### Tissue collection, harvesting and preparation

For biochemical analysis, tissue sampling was carried out according to Mably *et al.* (2015) with some modifications. Briefly, 2 days following the last injection, animals were sacrificed by cervical dislocation to avoid anaesthesia-mediated tau phosphorylation (Planel *et al.*, 2007) and intra-cardially perfused with ice-cold PBS using a 30-ml syringe to remove blood contamination. Brains were collected, the meninges were carefully removed and dissected hippocampi were immediately frozen on dry-ice and, then, stored at −80°C until use.

Hippocampal total protein lysates were carried out according to Castillo-Carranza *et al.* (2015) with some modifications. In details, frozen mice hippocampi were diced and homogenized in PBS with a protease inhibitor mixture (Roche) and 0.02% NaN_3_ using a 1:3 (w/v) dilution. Samples were then centrifuged at 10 000 rpm for 10 min at 4°C and the supernatants were collected.

Tris-buffered saline (TBS) extracts were carried out according to Mably *et al.* (2015) with some modifications. Frozen mice hippocampi were homogenized in five volumes (wt/vol) TBS, pH 7.4, plus proteases inhibitor cocktail (Sigma-Aldrich P8340) and phosphatase inhibitor cocktail (Sigma-Aldrich, Oakville, Ontario, Canada P5726/P2850) with 30 strokes of a glass Dounce tissue. The homogenate was centrifuged at 90 000 g at 4°C for 1 h. The supernatant (TBS extract) was removed and stored at −20°C.

Synaptosomes preparations were carried out as previously reported (Corsetti *et al.*, 2015; Florenzano *et al.*, 2017).

### Cloning, bacterial expression and purification of the 20–22 kDa NH_2_26-230 tau fragment (aka NH_2_htau)

cDNA fragment coding for the amino acids 26–230 of the isoform 4 of human tau protein (htau40) was cloned into the vector pET-11a (Novagen) suitable for the expression of recombinant proteins in BL21DE3 Gold *Escherichia coli* cells. After induction with IPTG, recombinant protein in lysates from bacterial pellet was purified to homogeneity by a two-step procedure: step 1 was a HiCood Q Sepharose 16/10; step 2 was Hitrap Phenyl 5 ml. Degree of protein purification was evaluated by Coomassie Brilliant Blue G-250 and checked by sodium dodecyl sulfate-polyacrylamide gel electrophoresis under reducing conditions by western blotting with commercial human-specific NH_2_-tau antibody (DC39N1 45–73aa) and with 12A12mAb (26–36aa). The molecular identity of purified peptide fraction was finally checked by electrospray ionization mass spectrometry.

### Detection of the NH_2_htau fragment by 12A12mAb-based enzyme-linked immunosorbent assay

High-binding black 96 well plates (Costar 3925, Corning, NY) were coated overnight at 4°C following the addition of 5 μg/ml 12A12mAb capture antibody diluted in coating buffer (50 mM NaHCO_3_, pH9.6). Plates were washed with PBST (PBS containing 0.05% Tween-20) and incubated with 5% non-fat dry milk (w/v) in PBST at room temperature (RT) for 2–4 h while shaking to block non-specific binding sites. Plates were washed with PBST and incubated (50 μl/well) overnight at 4°C while shaking with recombinant NH_2_26-230 tau fragment standard curves prepared in assay buffer concentration of 5% milk (w/v) and 0.05% Tween-20 (v/v) in PBS, pH 8. Plates were washed with PBST and incubated (50 μl/well) overnight at 4°C with rabbit H150 antibody (1–150aa; Santa-Cruz sc-5587) diluted to the final concentration of 2.5 μg/ml in assay buffer concentration of 5% milk and 0.05% Tween-20 (v/v) in PBS. Plates were then washed with PBST and added with 50 μl/well of rabbit horseradish peroxidase-conjugated secondary antibody for 1 h at RT. Plates were washed with PBST and developed at RT using TMB substrate (T0440; Sigma-Aldrich, Oakville, Ontario, Canada). Luminescence counts were measured using Packard TopCount (PerkinElmer, MA). Log-transformed luminescence counts from individual samples were interpolated to concentration using a second-order polynomial fit to the respective standards (GraphPad Prism 5.00, GraphPad Software, San Diego).

### Detection of i.v.-delivered 12A12mAb in TBS brain extracts

The concentration of i.v. delivered anti-tau 12A12mAb was measured in TBS brain extracts according to Mably *et al.* (2015) with some modifications. A solid-phase enzyme-linked immunosorbent assay (ELISA) was performed on the plate-immobilized synthetic NH_2_26-44aa which was used as catching antigenic peptide, being the minimal Alzheimer’s disease-relevant (Borreca *et al.*, 2018), active moiety of the parental longer NH_2_26-230 (Amadoro *et al.*, 2004, 2006). Clear 96 well high-binding plates (Costar 3925, Corning, NY) were coated (50 μl/well) of 5 μg/ml synthetic NH_2_26-44aa in coating buffer (0.05 M Carbonate-Bicarbonate, pH9.6) overnight at 4°C. Wells were washed twice with PBST and loaded (50 μl/well) with (i) the standard curve prepared by making serial dilutions of 12A12mAb (250–0.12 ng/ml), (ii) the TBS extracts diluted 1/50, 1/10, 1/2, 1/1.3 or (iii) blanks diluted in assay buffer concentration of 5% milk and 0.05% Tween-20 (v/v) in PBS overnight at 4°C. Plates were then washed with PBST and added with 50 μl/well of rabbit horseradish peroxidase-conjugated secondary antibody for 1 h at RT. Plates were washed with PBST and developed at RT using TMB substrate (T0440; Sigma-Aldrich, Oakville, Ontario, Canada). Luminescence counts were measured using Packard TopCount (PerkinElmer, MA). Log-transformed luminescence counts from individual samples were interpolated to concentration using a second-order polynomial fit to the respective standards (GraphPad Prism 7.00, GraphPad Software, San Diego).

### Cell culture, treatment and protein lysates preparation

SH-SY5Y human neuroblastoma cells were cultured and terminally differentiated into post-mitotic neurons according to Corsetti *et al.* (2008). Culture treatment and protein lysates preparation were carried out by using standard procedures, according to Borreca *et al.* (2018).

### Western blot analysis and densitometry

Western blot analysis and densitometry were carried out by using standard procedures, according to Borreca *et al.* (2018).

The following antibodies were used: anti-Aβ/APP 6E10 (4–9aa) mouse MAB1560 Chemicon; anti-Alzheimer precursor protein 22C11 (66–81aa of N-terminus) mouse APP-MAB348 Chemicon Temecula-CA; anti-pan tau protein H150 (1–150aa of N-terminus) rabbit sc-5587 Santa Cruz Biotechnology; anti-pan tau protein DC25(microtubule binding repeat) mouse T8201 Sigma-Aldrich; tau 21 (21–36aa of N-terminus) rabbit AHB0371 Biosource International (USA); anti-N-tau (45–73aa) DC39N1 mouse T8451 Sigma-Aldrich; neuronal marker β III tubulin antibody mouse ab78078 (clone 2G10) Abcam; GAPDH antibody (6C5) mouse sc-32233 Santa Cruz Biotechnology; activity-regulated cytoskeleton-associated protein (C-7) mouse sc-17839 Santa Cruz Biotechnology; glial fibrillary acidic protein antibody rabbit Z0334 Dako; Iba1 antibody rabbit Wako 016-20001 (for WB).

### Novel object recognition test

Two days after the last i.v. injection, mice run the novel object recognition (NOR) task (Antunes and Biala, 2012) to check the hippocampal-dependent episodic memory (Bevins and Besheer, 2006; Akkerman *et al.*, 2012*a*, *b*). The entire task was performed in three consecutive sessions during the same day (1-day version), according to previously published protocol (Borreca *et al.*, 2018).

### Object place recognition test

The object place recognition paradigm involves the activity of the hippocampus and is used to test the short-term memory (Vogel-Ciernia and Wood, 2014). The animals, which underwent the NOR paradigm with a training and test session, were tested in the object place recognition paradigm 24 h later, with a separated training and test session. The objects used for the object place recognition were different from those used previously for the NOR test in order to avoid possible confounding effects. The entire behavioural task including three phases (a common habituation phase, a training phase and a test phase) was performed by using standard protocol (Lesburguères *et al.*, 2017).

### Spontaneous alternation (Y-maze) test

Evaluation of short-term working memory was carried out by using the spontaneous alternation version of the Y-maze, which involves different brain structures ranging from the hippocampus to the prefrontal cortex. Y-maze testing also indicates overall activity, or hyperactivity, based on the number of arm entries Spontaneous alternation, expressed as a percentage, was calculated by dividing the number of entries into all three arms on consecutive choices (correct choices) by number of arm entries subtracted by two, then multiplying the quotient by 100 (Hiramatsu *et al.*, 1997; Wall and Messier, 2002). A high spontaneous alternation rate is indicative of sustained working memory because the animals must remember which arm was entered last to know not to re-enter it.

### Energy metabolism

Energy expenditure (EE) and oxygen consumption (VO_2_) were measured by an indirect calorimeter system (TSE PhenoMaster/LabMaster System^®^) in vehicle- or 12A12mAb-treated mice by a constant air flow of 0.35 l/min. Mice were adapted for 6 h to the metabolic chamber before the start of recording, and VO_2_ was measured every 30 min in each mouse, starting at 7:00 PM and ending automatically after 4 days (96 h later). RT was kept constant (22 ± 1°C). The EE for each sample point was evaluated across the 48 h of total recording. Locomotor activity was assessed during the indirect calorimetric assay by the number of infra-red beams broken. Each cage of the calorimeter system is equipped with the InfraMot^®^ device that uses ‘passive infrared sensors’ to detect and record the motor activity of the mouse by the body-heat image and its spatial displacement across time. Any type of body movement was detected and recorded as activity counts. EE was also analysed by considering animals’ steady conditions or lack of motor activity (resting EE, REE; only values between 0 and 2 activity counts were included).

### Golgi-Cox staining and dendritic spine analysis

Two days after the last i.v. injection, mice were sacrificed with a lethal dose of anaesthetic (Zoletil/Rompun 800 and 100 mg/kg, respectively) and perfused transcardially with 0.9% saline solution. Brains were dissected and immediately immersed in a Golgi-Cox solution (1% K_2_Cr_2_O_7_, 1% HgCl_2_ and 0.8% K_2_CrO_4_) at RT for 6 days, according to a previously described protocol (Gibb and Kolb, 1998; Rosoklija *et al.*, 2014). On the seventh day, brains were transferred in a 30% sucrose solution for cryoprotection and then sectioned with a vibratome. Staining and dendritic spine analysis was carried out according to standard criteria (Horner and Arbuthnott, 1991; Leuner *et al.*, 2003) and by using previously published method (Borreca *et al.*, 2018). Statistical comparisons were made on single mouse values obtained by averaging the number of spines counted on neurons of the same mouse.

### Electrophysiological recordings

Two days after the last i.v. injection of 12A12mAb, mice were anaesthetized by halothane or isofluorane inhalation and decapitated. The brain was rapidly removed and put in ice-cold cutting solution (in mM: 124 NaCl, 3.2 KCl, 1 NaH_2_PO_4_, 26 NaHCO_3_, 2 MgCl_2_, 1 CaCl_2_, 10 glucose, 2 sodium pyruvate and 0.6 ascorbic acid, bubbled with 95% O_2_–5% CO_2_, pH 7.4). Electrophysiological recordings were performed on hippocampal coronal slices (400 μm thick) by using standard procedures (Podda *et al.*, 2016; Nobili *et al.*, 2017).

### Statistical analysis

In box-and-whisker plots, the centre lines denoted median values, edges were upper and lower quartiles, whiskers showed minimum and maximum values and points were individual experiments. Other data were expressed as mean ± SEM. All data were representative of at least three separate experiments (*n* = independent experiments). Statistically significant differences were calculated by one-way or two-way analysis of variance (ANOVA) followed by Bonferroni’s, Fisher’s and Dunnett’s *post hoc* tests for multiple comparisons among more than two groups. *P* < 0.05 was accepted as statistically significant (*P < 0.05; **P < 0.01; ***P < 0.0005; ****P < 0.0001). All statistical analyses were performed using GraphPad Prism 7 software.

### Data availability

The datasets used and/or analysed during the current study and detailed protocols/experimental procedures are available from the corresponding author on reasonable request. Western blotting fill-size images can be found in Supplementary Materials.

## Results

### Intravenously injected anti-NH_2_htau 12A12mAb is biologically active in the animals’ hippocampus

Tg2576 and 3xTg mice—two well-established animal Alzheimer’s disease models (Hsiao *et al.*, 1996; Oddo *et al.*, 2003) which express the human APP695 with Swedish mutations (K670N-M671L), alone or in combination with MAPT P301L and PSEN1 M146V, respectively—were analysed because these Tg animals are recognized to display progressive tau-dependent, hippocampus-based synaptic and cognitive impairments (Oddo *et al.*, 2006; Castillo-Carranza *et al.*, 2015; Amar *et al.*, 2017). The hippocampal parenchyma was examined in the present study, since this vulnerable cerebral area: (i) selectively and disproportionally degenerates at early stages of mild cognitive impairment prior to the clinical diagnosis of full-blown dementia (Honer *et al.*, 1992; West *et al.*, 1994; Gomez-Isla *et al.*, 1996; Kordower *et al.*, 2001; Scheff *et al.*, 2006*a*, *b*); (ii) preferentially develops tau neuropathology into synaptic compartments, whose initial deterioration is considered the best correlate of cognitive decline in Alzheimer’s disease symptomatology by critically subserving the transition from normal aging to mild cognitive impairment (Braak and Braak, 1991, Arriagada *et al.*, 1992; Guillozet *et al.*, 2003; Markesbery *et al.*, 2006, 2010; Pooler *et al.*, 2014; Spires-Jones and Hyman, 2014).

**Figure 1:**
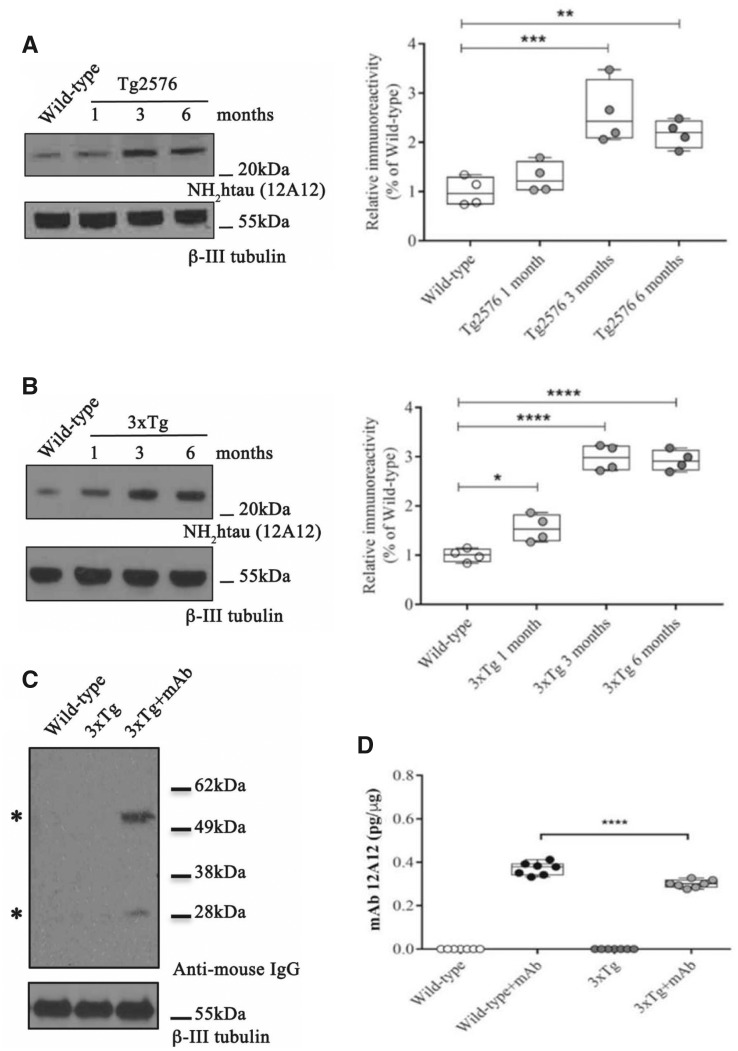
**The i.v.-injected 12A12mAb anti-tau antibody is biologically active into the animals’ hippocampus.** (**A**, **B**) Western blot analysis carried out on hippocampi from Tg2576 and 3xTg Alzheimer’s disease mice at different ages (1, 3 and 6 months old) and from 6-month-old WT by probing with 12A12mAb (left). β-III tubulin was used as loading control. Arrows on the right side indicate the molecular weight (kDa) of bands calculated from migration of standard proteins. Full uncropped blots are available in Supplementary Fig. 5. Pooled data and relative densitometric quantifications are reported on the right. In this and all other figures, in box-and-whisker plots the centre lines denote median values, edges are upper and lower quartiles, whiskers show minimum and maximum values and points are individual experiments. Statistically significant differences (see details in the main text) were calculated by ANOVA followed by *post hoc* test for multiple comparisons among more than two groups. *P* < 0.05 was accepted as statistically significant (*P < 0.05; **P < 0.01; ***P < 0.0005; ****P < 0.0001). (**C**) Western blotting analysis was carried out by probing with anti-mouse IgG as primary antibody (Thermo-Fisher 10400C) on hippocampal protein extracts (40μg) from animals of the three experimental groups (WT, ‘naïve’ 3xTg, 3xTg + mAb) which underwent i.v. injection with saline or 12A12mAb (see details in Materials and methods section). β-III tubulin was used as loading control. Arrows on the right side indicate the molecular weight (kDa) of bands calculated from migration of standard proteins. Full uncropped blots are available in Supplementary Fig. 5. Notice that 3xTg animals, which are systemically i.v. injected for 14 days with 12A12mAb (see details in Materials and methods section), exhibit high levels of cerebral mouse IgG when compared to not-vaccinated controls confirming that a fraction of mAb injected into the tail vein is present in the hippocampal parenchyma. Asterisks point to the light and heavy antibody chains (25 and 50 kDa, respectively). (**D**) Brain levels of anti-tau antibody 12A12mAb were evaluated by ELISA in the TBS-soluble fraction of hippocampal homogenates from WT and 3xTg mice that i.v. received saline or 12A12mAb for 14 days (see details in the Materials and methods section). The ELISA used to measure the anti-tau antibody relies on the plate-immobilized recombinant NH_2_26-44aa tau which, being the minimal Alzheimer’s disease-relevant (Borreca *et al.*, 2018) active moiety of the parental longer NH_2_26-230 (Amadoro *et al.*, 2004, 2006), was used as catching peptide. Notice that a significant portion of the 12A12mAb in 3xTg brains is bound to endogenous NH_2_htau and does non-specifically interact with the large amount of intracellular tau released during homogenization. Statistically significant differences (see details in the main text) were calculated by ANOVA followed by *post hoc* test for multiple comparisons among more than two groups. *P* < 0.05 was accepted as statistically significant.

Before addressing the possible benefits offered by systemic delivery of the cleavage-specific 12A12mAb (Δ-_25_NH_2_tau(Q26–36aa)-terminus), we determined an appropriate lifetime at which Tg-Alzheimer’s disease mice could be employed for antibody immunization experiments. In light of these considerations, the *in vivo* level of the pathogenic 20–22 kDa NH_2_htau was measured by western blotting sodium dodecyl sulphate-polyacrylamide gel electrophoresis analyses carried out on synaptosomal preparations from hippocampi of WT and Alzheimer’s disease Tg animals of both genetic backgrounds at three ages (1, 3, 6-month-old Tg2576 and 3xTg). As shown in Fig. 1A and B, the signal intensity of 12A12mAb-positive NH_2_htau immunoreactivity band was virtually undetectable in 6-month-old cognitively intact controls but appeared to be up-regulated in diseased animals (one-way ANOVA followed by Dunnett’s *post hoc* test for multiple comparisons Tg2576 *F*(3,12) = 13.34, *P* = 0.0004; 3-month-old Tg2576 versus 6-month-old WT, ***P < 0.0005; 6-month-old Tg2576 versus 6-month-old WT, **P < 0.01; 3xTg *F*(3,12) = 76.79, *P* < 0.0001; 1-month-old 3xTg versus 6-month-old WT, *P < 0.05; 3-month-old 3xTg versus 6-month-old WT, ****P < 0.0001; 6-month-old 3xTg versus 6-month-old WT, ****P < 0.0001). Consistent with previous investigations from rodent preparations (Rohn *et al.*, 2002; Corsetti *et al.*, 2008) and human nerve terminals specimens (Amadoro *et al.*, 2010, 2012; Corsetti *et al.*, 2015; Sokolow *et al.*, 2015), the steady-state expression level of the neurotoxic 20–22 kDa NH_2_htau truncated fragment significantly increased and time-dependently accumulated starting from 1 month of age into synaptic-enriched fractions of cognitively impaired older animals from both Alzheimer’s disease Tg mouse models. The specific ability of 12A12mAb in binding the 20–22 kDa NH_2_htau fragment *in vitro*, both in recombinant and native forms, was checked by western blotting and ELISA (Supplementary Fig. 1).

Having ascertained that the NH_2_htau accumulated into hippocampal synapses under pathological conditions in association with progressive disruption of animals’ memory/learning function(s), we investigated whether the 12A12mAb could be exploited to systemic tau-directed immunization regimen. In particular, we ascertained whether 12A12mAb was able to gain access to the cerebral parenchyma after its peripheral administration, an optimal prerequisite for local engagement of the pathogenic target and its successful neutralization/clearance *in vivo*. To this aim, 6-month-old mice from these two different strains (Tg-Alzheimer’s disease) were infused over 14 days with two weekly injections of 12A12mAb (30 μg/dose) administered on two alternate days to the lateral vein of the tail. Both age-matched WTs and ‘naïve’ (i.e. not-immunized) Tg Alzheimer’s disease counterparts, which were sham-infused under the same experimental conditions with vehicle (saline) only, were also included as negative controls. By probing with anti-mouse IgG used as primary antibody, western blotting analysis (Fig. 1C) carried out on hippocampal protein extracts from the three experimental groups (WT, ‘naïve’ Tg-Alzheimer’s disease, Tg-Alzheimer’s disease + mAb which, importantly, were sacrificed and thoroughly perfused with PBS in order to make sure that their brains were free of blood contaminations) showed that 12A12mAb-injected 3xTg animals exhibited high levels of cerebral mouse IgG when compared to not-vaccinated, saline-treated controls. This qualitative finding is in line with previous reports on the ability of other, intravenously administered anti-tau antibodies to cross the blood–brain barrier of diseased Tg mice (about 0.1% of delivered total amount), likely owing to its age-related impairment and increased permeability (Asuni *et al.*, 2007; Blair *et al.*, 2015; Mably *et al.*, 2015; Bennett *et al.*, 2018).

Next, to confirm that peripherally delivered 12A12mAb was actually able to bind the NH_2_htau *in vivo*, we carried out ELISA quantitative test on TBS-soluble fractions isolated from hippocampi of WT, ‘naïve’3xTg and 3xTg + mAb animals after 14 days i.v. injection. Healthy, WT mice infused with 12A12mAb under the same experimental conditions (WT + mAb) were also included to ascertain whether 12A12mAb could enter the brain from periphery despite the intact blood–brain barrier and/or the lack of tau pathology into the CNS. It’s worth underlying that: (i) the ELISA test aimed at assessing the cerebral amount of injected 12A12mAb is based on the plate-immobilized synthetic NH_2_26-44aa which, being the minimal Alzheimer’s disease-relevant (Borreca *et al.*, 2018) active moiety of the parental longer NH_2_26-230 (Amadoro *et al.*, 2004, 2006), was used as catching peptide; (ii) only the free (i.e. unoccupied) antibody can readily bind to its immobilized specific antigen and be measured, whereas the tau-bound antibody is not detectable. As shown in Fig. 1D, a sizeable proportion of the injected 12A12mAb was unbound and biologically active (antigen-binding competent) in 3xTg brains, being able to recognize the synthetic plate-immobilized recombinant tau peptide. Interestingly, the levels of i.v.-administered 12A12mAb were significantly lower in 3xTg + mAb experimental group than in WT + mAb counterpart (two-way ANOVA analysis followed by Bonferroni’s *post hoc* test for multiple comparisons, genotype × treatment interaction *F*(1,24) = 28.92, *P* < 0.0001; WT + saline versus Tg-Alzheimer’s disease + saline *n*.s., *P* > 0.99; ****P < 0.0001 for all other pair comparisons), indicating that a higher fraction of this antibody is actually bound *in vivo* to the endogenously generated NH_2_htau antigen—and thus less available for capture in *in vitro* ELISA assay—into the hippocampi from diseased animals than in healthy controls. Similar results were also found in 6-month-old Tg2576 animals from the other genetic background which were analysed and treated under the same experimental conditions (data not shown).

Collectively, these findings demonstrated that: (i) the pathological tau truncated at its N-terminal domain early accumulates into hippocampal synapses from Tg-Alzheimer’s disease, suggesting that it might contribute to the age-dependent disruption of animals’ memory and learning functions; (ii) after its i.v. injection, 12A12mAb can be actively up-taken into the brain because an appreciable percentage of free and antigen-binding competent form of antibody is present into the hippocampus both from healthy controls and 3xTg immunized mice, regardless of the integrity of their blood–brain barrier and/or the presence of tau pathology; (iii) 12A12mAb does not aspecifically interact, neither in WT nor in 3xTg, with the large amount of intracellular full-length normal tau which is routinely released during procedure of samples homogenization, in line with our previous *in vivo* observations advocating its cleavage-specificity towards the NH_2_htau truncated fragment (Amadoro *et al.*, 2012, Supplementary Fig. 1); (iv) 12A12mAb is not in limiting amount and, thus, endowed with potential therapeutic effect (*in vivo* target-engagement) because after immunization it is locally detectable in its active/antigen-competent state into mouse brains, with higher level in the WT controls than in diseased 3xTg ones.

### 12A12mAb passive immunization reduces both pathological tau and APP/Aβ metabolisms into synaptic compartments from treated Alzheimer’s disease Tg mice at the prodromal stage of neuropathology

Co-occurrence between tau and Aβ pathology has been described to take place within neuronal processes and nerve ending compartments at early stages of Alzheimer’s disease (Takahashi *et al.*, 2010; Amadoro *et al.*, 2012; Spires-Jones and Hyman, 2014; Rajmohan and Reddy, 2017). In the preclinical models of Tg2576 and 3xTg, Aβ exerts its synaptotoxicity, at least in part, via tau, but both separate and synergistic neurodegenerative mechanisms have been also described in these two experimental paradigms (Nisbet *et al.*, 2015; Li and Gotz, 2017; Polanco *et al.*, 2018). Recent *in vitro* and *in vivo* evidence have demonstrated that Aβ and tau pathology—in addition to their direct and/or indirect interaction (Castillo-Carranza *et al.*, 2015; Dai *et al.*, 2017, 2018; Rajamohamedsait *et al.*, 2017)—can damage the synaptic terminals in an APP-dependent manner suggesting that its increased expression level *per se* should be considered as an additional therapeutic target to preserve the integrity and function of crucial neuronal networks (Gulisano *et al.*, 2018; Kametani and Hasegawa, 2018; Schreurs *et al.*, 2018).

**Figure 2:**
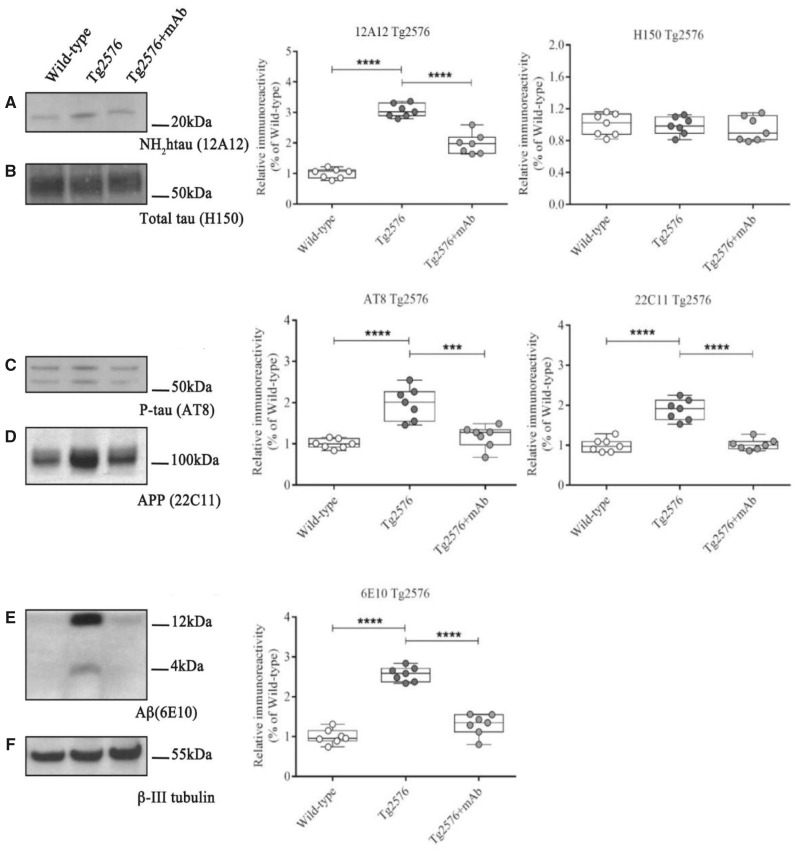
**Reduction of the hippocampal NH_2_htau in Tg-Alzheimer’s disease (Tg2576) mice immunized with 12A12mAb ameliorates the disease-associated synaptic neuropathology.** Representative blots (*n* = 5) of sodium dodecyl sulphate-polyacrylamide gel electrophoresis western blotting analysis (left) on isolated synaptosomal preparations from hippocampal region of animals from three experimental groups (WT, Tg-Alzheimer’s disease and Tg-Alzheimer’s disease + mAb) of Tg2576 strain to assess the content of the NH_2_htau fragment (**A**), total tau full-length (**B**), AT8-phosphorylated tau (**C**), APP holoprotein (**D**) and Aβ monomeric and oligomeric species (**E**). Data were quantified for molecular weight size ranges for each antibody and normalized to β-III tubulin which was used as loading control (**F**) and relative densitometric quantifications are reported (right). Arrows on the right side indicate the molecular weight (kDa) of bands calculated from migration of standard proteins. Full uncropped blots are available in Supplementary Fig. 6. Notice that changes in levels of total tau are not statistically significant. Statistically significant differences (see details in the main text) were calculated by ANOVA followed by *post hoc* test for multiple comparisons among more than two groups. *P* < 0.05 was accepted as statistically significant (*P < 0.05; **P < 0.01; ***P < 0.0005; **** *P* < 0.0001).

**Figure 3:**
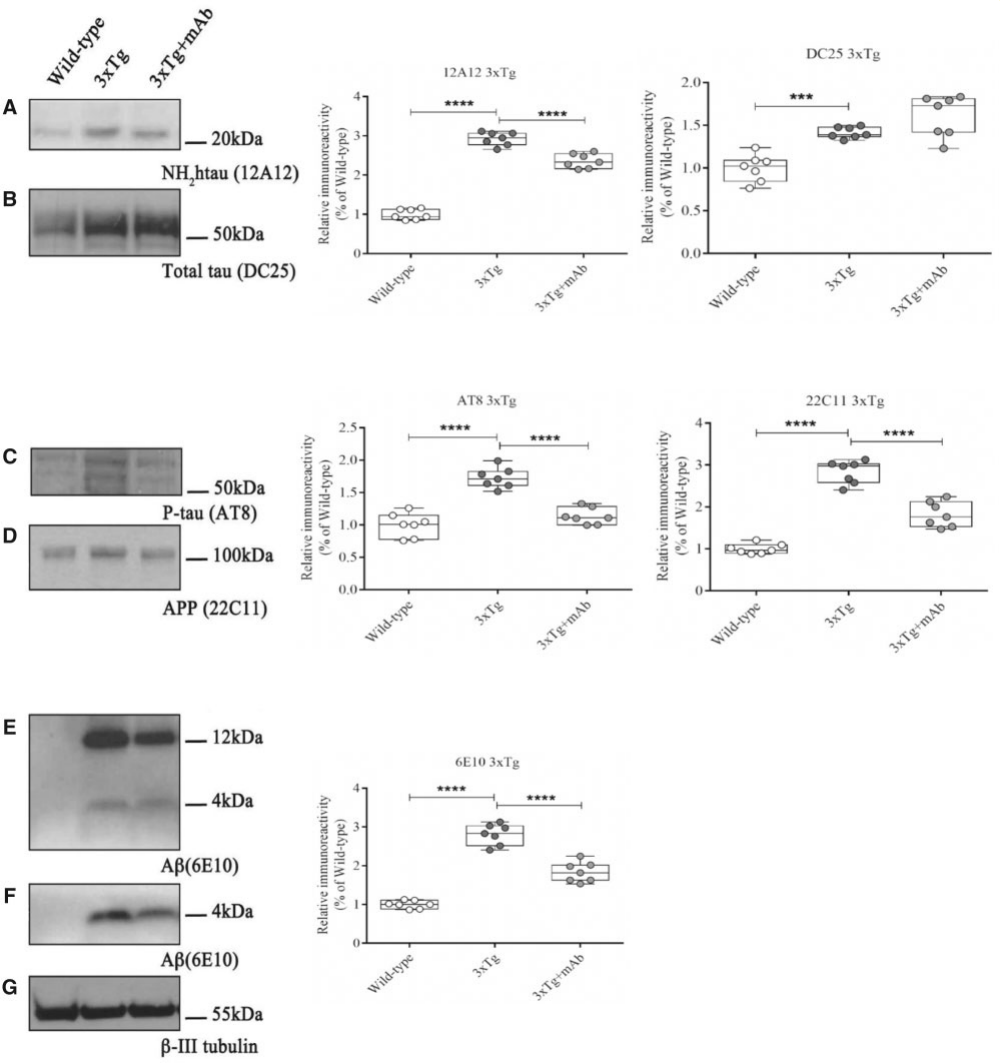
**Reduction of the hippocampal NH_2_htau in Tg-Alzheimer’s disease mice (3xTg) immunized with 12A12mAb ameliorates the disease-associated synaptic neuropathology.** (**A–G**)Representative blots (*n* = 5) of sodium dodecyl sulphate-polyacrylamide gel electrophoresis western blotting analysis (left) on isolated synaptosomal preparations from hippocampal region of animals from three experimental groups (WT, Tg-Alzheimer’s disease and Tg-Alzheimer’s disease + mAb) of 3xTg strain to assess the content of the NH_2_htau fragment (**A**), total tau full-length (**B**), AT8-phosphorylated tau (**C**), APP holoprotein (**D**) and Aβ monomeric and oligomeric species (**E**/**F**). Data were quantified for molecular weight size ranges for each antibody and normalized to β-III tubulin which was used as loading control (**G**) and relative densitometric quantifications are reported (right). Arrows on the right side indicate the molecular weight (kDa) of bands calculated from migration of standard proteins. Full uncropped blots are available in Supplementary Fig. 7. Notice that changes in levels of total tau are not statistically significant. Statistically significant differences (see details in the main text) were calculated by ANOVA followed by *post hoc* test for multiple comparisons among more than two groups. *P* < 0.05 was accepted as statistically significant (*P < 0.05; **P < 0.01; ***P < 0.0005; ****P < 0.0001).

In view of these considerations, we investigated whether the antibody-mediated neutralization of pathogenic NH_2_-truncated tau following i.v. 12A12mAb infusion could mitigate *in vivo* the occurrence of neurochemical derivatives from the abnormal APP and tau metabolisms which are largely recognized to compromise the Alzheimer’s disease nerve terminals at prodromal disease stages (Braak and Del Tredici, 2015). To this aim, western blotting sodium dodecyl sulphate-polyacrylamide gel electrophoresis analyses (Fig. 2, Tg2576; Fig. 3, 3xTg) were carried out on hippocampal synaptosomal preparations from the three experimental groups (WT, ‘naïve’ Tg-Alzheimer’s disease, Tg-Alzheimer’s disease + mAb) of both genetic backgrounds (3-month-old Tg2576 and 3xTg) by probing with both 12A12mAb and specific commercial antibodies detecting the Alzheimer’s disease-like, site-specific tau hyperphosphorylation at Ser202/Thr205 epitope (AT8) (Goedert *et al.*, 1995) and the accumulation of soluble 6E10-positive APP/Aβ derivatives (Teich *et al.*, 2015). As shown in Figs 2A and 3A—and in line with Fig. 1A and B—the intensity signal of the neurotoxic 20–22 kDa NH_2_htau truncated fragment was markedly increased in saline-treated, ‘naïve’ Tg-Alzheimer’s disease mice when compared to non-Tg WT controls (one-way ANOVA followed by Bonferroni’s *post hoc* test for multiple comparisons *F*(2,18) = 117.5, *P* < 0.0001, Tg2576; *F*(2,18) = 34.54, *P* < 0.0001, 3xTg; ****P < 0.0001, Tg2576 versus WT; ****P < 0.0001, 3xTg versus WT). Importantly, the 12A12mAb treatment significantly reduced the synaptic load of 20–22 kDa NH_2_htau form(s) in Tg-Alzheimer’s disease animals from both strains indicating that this antibody, after i.v. delivery, successfully engaged/intercepted its target into hippocampus with consequent neutralization/clearance *in vivo* (one-way ANOVA followed by Bonferroni’s *post hoc* test ****P < 0.0001, Tg2576 + mAb versus Tg2576; ****P < 0.0001, 3xTg + mAb versus 3xTg). Following 12A12mAb immunization, the AT8 immunoreactivity was strongly inhibited in Tg-Alzheimer’s disease animals (one-way ANOVA followed by Bonferroni’s *post hoc* test *F*(2,18) = 23.72, *P* < 0.0001, Tg2576; *F*(2,18) = 42.18, *P* < 0.0001, 3xTg; Tg2576 versus WT, ****P < 0.0001; Tg2576 + mAb versus WT n.s., *P* = 0.7913; Tg2575 + mAb versus Tg2576, ***P < 0.0005; 3xTg versus WT, ****P < 0.0001; 3xTg + mAb versus WT n.s., *P* = 0.3747; 3xTg + mAb versus 3xTg, ****P < 0.0001), proving that the anti-truncated tau antibody was able to down-regulate the extent of tau neuropathology *in vivo* (Figs 2C and 3C). A drastic decline and/or disappearance of the expression levels of 6E10-positive APP/Aβ specie(s) (i.e. 4 kDa Aβ monomer, 14 kDa low-molecular weight Aβ oligomers or APP C-terminal fragment (βCTF)) was also clearly observed in Tg-Alzheimer’s disease hippocampal synapses from treated experimental groups of both genetic backgrounds (Figs 2E and 3E and F) (one-way ANOVA followed by Bonferroni’s *post hoc* test *F*(2,18) = 104.7, *P* < 0.0001, Tg2576; *F*(2,18) = 115.8, *P* < 0.0001, 3xTg; Tg2576 versus WT, ****P < 0.0001; Tg2576 + mAb versus WT n.s., *P* = 0.0536; Tg2576 + mAb versus Tg2576, ****P < 0.0001; ****P < 0.0001 for all pair comparisons from 3xTg). Importantly, the steady-state expression level of total tau detected by probing with H150 and DC25 (Figs 2B and 3B), two commercial anti-pan tau antibodies binding both murine and human tau isoforms (Zilka *et al.*, 2006; Lee *et al.*, 2010; Um *et al.*, 2011; Schroeder *et al.*, 2016), was unchanged in synapses from Alzheimer’s disease Tg animals after 12A12mAb immunization regimen, with significantly higher level of total tau detected in 3xTg in comparison with WT controls due to the presence of both endogenous and human Tg proteins (one-way ANOVA followed by Bonferroni’s *post hoc* test *F*(2,18) = 0.3014, *P* = 0.7434 Tg2576; *F*(2,18) = 22.8 *P* < 0.0001 3xTg; n.s. *P* > 0.999 for all pair comparisons from Tg2576; 3xTg versus WT ***P < 0.0005; 3xTg + mAb versus WT ****P < 0.0001; 3xTg + mAb versus 3xTg n.s. *P* = 0.1577). These findings are consistent with tau cleavage-specificity of 12A12mAb which selectively binds *in vivo* the neurotoxic NH_2_htau truncated specie(s) (Amadoro *et al.*, 2012; Corsetti *et al.*, 2015) without showing any cross-reaction towards the full-length form of protein (Supplementary Fig. 1). Finally, as visualized by 22C11 commercial antibody (Figs 2D and 3D), 12A12mAb immunization resulted to act upstream on Aβ production by normalizing the disease-associated up-regulation in the expression level of APP full-length holoprotein in both Tg Alzheimer’s disease mice models (one-way ANOVA followed by Bonferroni’s *post hoc* test *F*(2,18) = 46.07 *P* < 0.0001 Tg2576; *F*(2,18) = 97.33 *P* < 0.0001 3xTg; Tg2576 versus WT ****P < 0.0001; Tg2576 + mAb versus Tg2576 ****P < 0.0001; Tg2576 + mAb versus WT n.s. *P* = 0.999; ****P < 0.0001 3xTg for all comparisons). Interestingly, this evidence supports more recent studies which suggest a prominent, causal role of APP accumulation in triggering synaptotoxicity and tauopathy (Kametani and Hasegawa, 2018; Schreurs *et al.*, 2018).

Collectively, these results demonstrate that: (i) when i.v. administrated to young (3 months old) Tg2576 and 3xTg mice—two well-established Alzheimer’s disease animal models showing tau-dependent neuropathology (Oddo *et al.*, 2006; Castillo-Carranza *et al.*, 2015; Amar *et al.*, 2017)—the cleavage-specific 12A12mAb is able to reach an appreciable concentration into the hippocampal parenchyma ensuing an effective binding/degradation of the pathologic 20–22 kDa NH_2_htau form(s); (ii) the *in vivo* antibody-mediated removal of the 20–22 kDa NH_2_htau form(s) alleviates the detrimental alterations of both APP/Aβ and tau metabolisms (i.e. AT8 tau hyperphosphorylation, APP/Aβ species accumulation and processing) commonly occurring at the earliest stage of Alzheimer’s disease progression into nerve endings; (ii) the 12A12mAb-mediated immunodepletion of the toxic 20–22 kDa NH_2_htau form(s) takes place in the absence of any significant change in the stability/turnover of normal full-length tau protein which is known to be endowed with important physiological functions into synaptic compartments (Pooler *et al.*, 2014; Regan *et al.*, 2017) and whose reduction, even if partial, is extremely harmful in terminally differentiated post-mitotic neurons *in vivo* (Biundo *et al.*, 2018; Velazquez *et al.*, 2018).

### Cognitive performance is significantly improved in symptomatic Alzheimer’s disease Tg mice after i.v. 12A12mAb delivery

**Figure 4:**
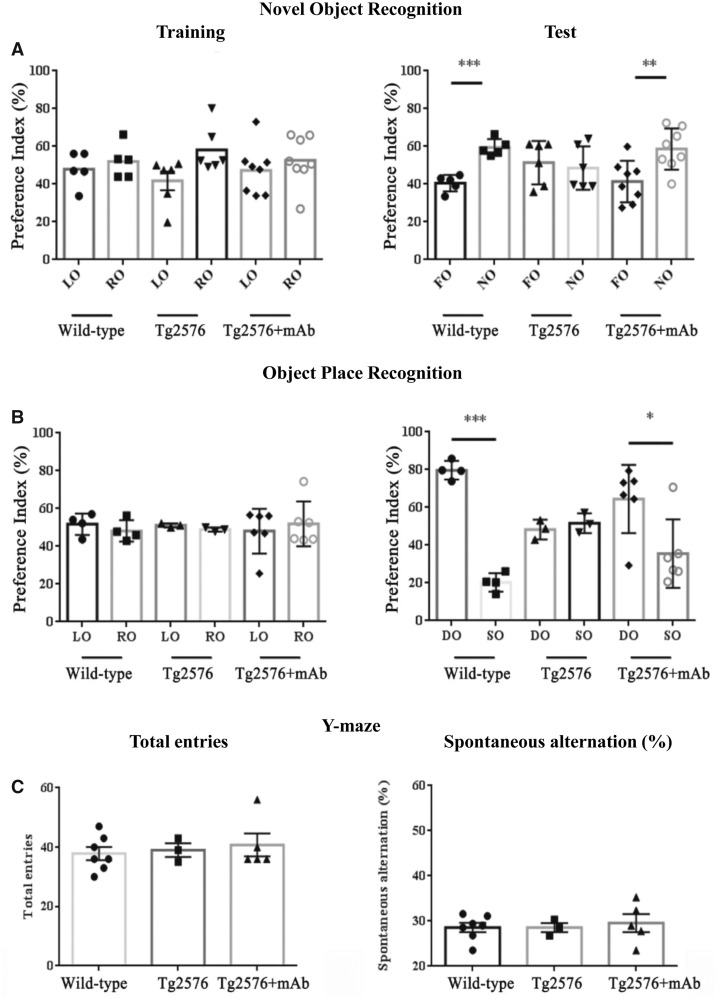
**Improved cognition in Tg-Alzheimer’s disease (Tg2576) mice immunized with 12A12mAb.** (**A–C**) Fourteen days after i.v. 12A12mAb immunization, the *in vivo* effect of NH_2_htau removal on cognitive performance was investigated in animals from the three experimental groups (WT, Tg-Alzheimer’s disease and Tg-Alzheimer’s disease + mAb) of Tg2576 genetic background in the NOR (**A**), OPR (**B**) and Y-maze (**C**) tasks (top to bottom). For NOR (**A**) and OPR (**B**): right and left histograms, respectively, represent the PI (%) of corresponding values measured during the test trial among animals from the different experimental groups (WT, Tg-Alzheimer’s disease and Tg-Alzheimer’s disease + mAb) of Tg2576 genetic background. The columns refer to objects presented during training and test trial. Analysis of PI (%) measured as time spending in the exploration of the novel/DO/(time spending in the exploration of novel/DO + time spending in the exploration of familiar/SO) × 100. Data were expressed as means ± SEM (*n* = 6–10). Values are means of at least three independent experiments and statistically significant differences (see details in the main text) were calculated by ANOVA followed by *post hoc* test for multiple comparisons among more than two groups. *P* < 0.05 was accepted as statistically significant (*P < 0.05; **P < 0.01; ***P < 0.0005; ****P < 0.0001). For Y-maze (**C**): right and left histograms, respectively, represent the total entries (the total arm entries correspond to the total number of arms entered) and the spontaneous alternation (the number of alternations corresponds to the successive entries into three different arms in overlapping triplet sets) of animals from the different experimental groups (WT, Tg-Alzheimer’s disease and Tg-Alzheimer’s disease + mAb) of Tg2576 genetic background. The percentage alternation was calculated as the ratio between number of correct triplets (e.g. ABC) and total entrances minus 2, multiplied by 100. Values are means of at least three independent experiments and statistically significant differences (see details in the main text) were calculated by ANOVA followed by *post hoc* test for multiple comparisons among more than two groups. *P* < 0.05 was accepted as statistically significant (*P < 0.05; **P < 0.01; ***P < 0.0005; ****P < 0.0001). FO = familiar object; LO = left object; OPR = object place recognition; RO = right object.

**Figure 5:**
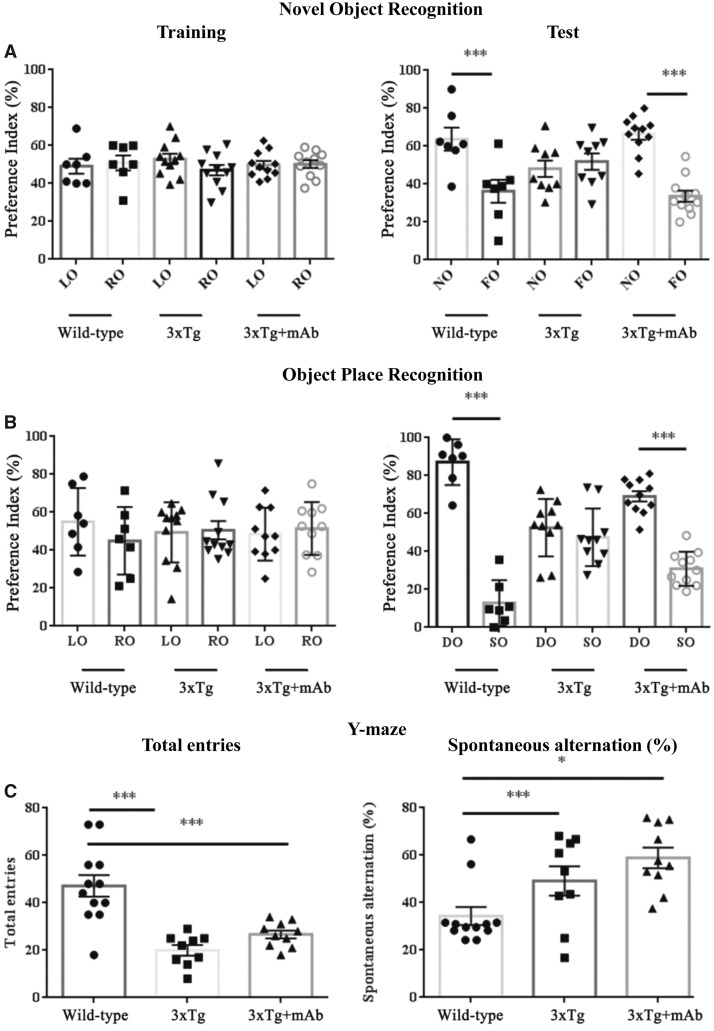
**Improved cognition in Tg-Alzheimer’s disease (3xTg) mice immunized with 12A12mAb.** Fourteen days after i.v. 12A12mAb immunization, the *in vivo* effect of NH_2_htau removal on cognitive performance was investigated in animals from the three experimental groups (WT, Tg-Alzheimer’s disease and Tg-Alzheimer’s disease + mAb) of 3xTg genetic background in the NOR (**A**), OPR (**B**) and Y-maze (**C**) tasks(top to bottom). For NOR (**A**) and OPR (**B**): right and left histograms, respectively, represent the PI (%) of corresponding values measured during the test trial among animals from the different experimental groups (WT, Tg-Alzheimer’s disease and Tg-Alzheimer’s disease + mAb) of 3xTg genetic background. The columns refer to objects presented during training and test trial. Analysis of PI (%) measured as time spending in the exploration of the novel/DO/(time spending in the exploration of novel/DO + time spending in the exploration of familiar/SO) × 100. Data were expressed as mean ± SEM (*n* = 6–10). Values are means of at least three independent experiments and statistically significant differences (see details in the main text) were calculated by ANOVA followed by *post hoc* test for multiple comparisons among more than two groups. *P* < 0.05 was accepted as statistically significant (*P < 0.05; **P < 0.01; ***P < 0.0005; ****P < 0.0001). For Y-maze (**C**): right and left histograms, respectively, represent the total entries (the total arm entries correspond to the total number of arms entered) and the spontaneous alternation (the number of alternations corresponds to the successive entries into three different arms in overlapping triplet sets) of animals from the different experimental groups (WT, Tg-Alzheimer’s disease and Tg-Alzheimer’s disease + mAb) of 3xTg genetic background. The percentage alternation was calculated as the ratio between number of correct triplets (e.g. ABC) and total entrances minus 2, multiplied by 100. Values are means of at least three independent experiments and statistically significant differences (see details in the main text) were calculated by ANOVA followed by *post hoc* test for multiple comparisons among more than two groups. *P* < 0.05 was accepted as statistically significant (*P < 0.05; **P < 0.01; ***P < 0.0005; ****P < 0.0001). FO = familiar object; LO = left object; OPR = object place recognition; RO = right object.

Having established that classical molecular determinants underlying the phenotypic Alzheimer’s disease manifestations are strongly reduced at early/pre-symptomatic stages of neuropathology following peripheral administration of 12A12mAb, cognitive functioning of symptomatic Tg-Alzheimer’s disease animals (6-month-old Tg2576 and 3xTg) was analysed under the same schedule of treatment by means of a comprehensive behavioural test battery (Fig. 4, Tg2576; Fig. 5, 3xTg).

The NOR task is a paradigm which is considered an appropriate readout for measures of learning/memory impairment in Tg and non-Tg animal models of tauopathies, including Alzheimer’s disease (Polydoro *et al.* 2009; Lanté *et al.*, 2015). Relevantly, the NOR behavioural task: (i) involves brain areas such as transentorhinal/entorhinal/perirhinal cortices and hippocampus which are pathologically relevant in this field, being affected by neurofibrillary tau changes at early stages of disease (Braak and Braak, 1991; Lasagna-Reeves *et al.*, 2011, 2012; Bengoetxea *et al.*, 2015; Sankaranarayanan *et al.*, 2015); (ii) is a non-aversive learning paradigm, avoiding the potential confounds of using differential rewards or punishments, able to evaluate the hippocampal-dependent episodic memory (Antunes and Biala, 2012; Leger *et al.*, 2013) which is the first type of memory affected in Alzheimer’s disease patients (Reed *et al.*, 1997; Zola and Squire, 2001; de Toledo-Morrell *et al.*, 2007; Salmon and Bondi, 2009; Grayson, *et al.*, 2015). Owing to innate and spontaneous preference of mice towards novelty, any increase in exploration of the novel object (NO) during the test session is to be ascribed to the animal’s ability in discriminating it from the familiar one (FO). This parameter was quantified as preference index (PI), which is calculated as the percentage of time spent exploring the new object over the total time spent exploring the two objects. In the recognition session, a PI for the NO ˃50% indicated that the NO was preferred, ˂50% that the FO was preferred and at 50% that no object was preferred (Hammond *et al.*, 2004).

Interestingly (Figs 4A and 5A), Tg2576 and 3xTg mice receiving 12A12mAb showed a significant rescue in short-term memory deficits when tested in this hippocampal-dependent task, being able to distinguish NO from FO (Tg2576 + mAb PI = 58.6%; 3xTg + mAb PI = 66.41%) just as WT, healthy non-Tg mice (B6SJL PI = 59.44%; C57 PI = 68.0%, respectively). On the other hand, saline-treated/‘naïve’ Tg-Alzheimer’s disease mice from both strains exhibited a poor performance when evaluated in NOR test because they spent the same time in exploring the NO versus the FO (Tg2576 PI = 48.51%; 3xTg PI = 50.48%, respectively). Accordingly, a two-way ANOVA of behavioural data (treatment × object discrimination) indicated significant difference between the three experimental groups of both strains (*F*(1,32) = 6.60 *P* = 0.01 for Tg2576; *F*(2,56) = 3.4 *P* = 0.04 for 3xTg) with the NO being preferred from 12A12mAb-infused Alzheimer’s disease Tg animals (Fisher’s *post hoc* test NO versus FO Tg2576 + mAb: **P < 0.01; 3xTg + mAb: ***P < 0.0005) which behaved in the same manner of WT, non-Tg ones (Fisher’s *post hoc* test NO versus FO B6SJL: ***P < 0.0005; C57: ***P < 0.0005). Conversely, not-immunized Alzheimer’s disease mice from both genetic backgrounds did not discriminate between NO and FO object and displayed defective mnestic abilities without any preference for NO (Fisher’s *post hoc* test Tg2576: *P* = 0.61; 3xTg: *P* = 0.32). Furthermore, these results were not due to an intrinsic inability of animals to interact with the objects because no significant difference (treatment × object discrimination) was measured during training phase among the animals’ cohorts from both strains which explored both objects for the same length of time and without any particular preference towards a side of the cage (two-way ANOVA analysis *F*(2,32) = 0.087 *P* = 0.916 for Tg2576 background; *F*(2,52) = 1.09: *P* = 0.34 for 3xTg mice; Fisher’s *post hoc* test left object versus right object B6SJL: *P* = 0.53, Tg2576: *P* = 0.20, Tg2576 + mAb: *P* = 0.30; Fischer’s *post hoc* test left object versus right object C57: *P* = 0.72, 3xTg: *P* = 0.91, 3xTg + mAb: *P* = 0.11).

In addition to the objects’ recognition memory, the hippocampal formation is devoted to store information about places in the organism’s environment, their spatial relations and the existence of specific objects in specific places (spatial memory) (O’Keefe and Conway, 1978; Broadbent *et al.*, 2004; Manns and Eichenbaum, 2009). Accordingly, immunized and not-immunized animals from the three experimental groups run the object place recognition task, another hippocampal-dependent paradigm which examines the memory/learning ability of mice in spatial relationships, rather than in objects recognition, by calculating the time spent in discriminating the spatially displaced ‘old familiar’ object relative to the stationary ‘old familiar’ object (Antunes and Biala, 2012; Vogel-Ciernia and Wood, 2014). Rodents normally display a clear preference for the object moved to a novel place [displaced object (DO)] in comparison to the object that remains in the same (familiar) place [stationary object (SO)], which confirms their proficiency for remembering which spatial locations have or have not been engaged earlier (Warburton *et al.*, 2013).

Again (Figs 4B and 5B), cognitive impairment of mice from the two genetic backgrounds (Tg2576 and 3xTg) was relieved following i.v. 12A12mAb injection because immunized animals were able to distinguish DO from SO (Tg2576 PI = 73.26%; 3xTg PI = 69.07%) by performing in spatial novelty memory task just as WT, healthy non-Tg ones (B6SJL PI = 79.71%; C57 PI = 71.48%, respectively). On the other hand, saline-treated ‘naïve’ Alzheimer’s disease Tg mice showed no preference for the moved object as they spent nearly equivalent amounts of time exploring the DO and SO which confirms that these not-immunized animals from both strains have object location memory dysfunction (Tg2576 home-cage PI = 48.29%; 3xTg home-cage PI = 52.53%, respectively). Consistently, a two-way ANOVA of behavioural data (treatment × object discrimination) indicated significant difference between the three animals’ cohorts in both strains analysed (*F*(2,20) = 9.68 *P* = 0.001 for Tg2576; *F*(2,50) = 33.11 *P* < 0.001 for 3xTg) with the DO being preferred from 12A12mAb-immunized Alzheimer’s disease mice (Fisher’s *post hoc* test DO versus SO Tg2576 + mAb: *P < 0.05, 3xTg + mAb: ***P < 0.0005) which behaved in the same manner of WT, non-Tg ones (Fisher’s *post hoc* test DO versus SO B6SJL: ***P < 0.0005, C57: ***P < 0.0005). In contrast, ‘naïve’ Tg2576 and 3xTg mice displayed no difference between DO and SO object with no obvious preference for DO (Fisher’s *post hoc* test Tg2576: *P* = 0.76; 3xTg: *P* = 0.35). Besides, regardless of the genetic background, no variation (treatment × object discrimination) was measured during the training phase among the three experimental groups which explored both objects for the same length of time and without any particular preference towards a side of the cage (two-way ANOVA analysis *F*(2,20) = 0.47 *P* = 0.63 for Tg2576 background; *F*(2,52) = 0.79 *P* = 0.46 for 3xTg mice; Fisher’s *post hoc* test left object versus right object B6SJL: *P* = 0.58, Tg2576: *P* = 0.76, Tg2576 + mAb: *P* = 0.47; Fischer’s *post hoc* test left object versus right object C57: *P* = 0.24, 3xTg: *P* = 0.86, 3xTg + mAb: *P* = 0.68).

After assessing the object discrimination and spatial memory, we also tested mice in the spontaneous alternation by employing the Y-maze, an hippocampal-dependent episodic-like behavioural test for measuring their willingness to explore new environments (exploratory tendency). Animals are started from the base of the Y-shaped apparatus placed horizontally and allowed to freely explore all three arms. The number of arm entries and the number of triads are recorded in order to calculate the percentage of alternation (Deacon and Rawlins, 2006; Borchelt and Savonenko, 2008) which is based on the fact that the rodent tends to choose the arm not visited before, reflecting memory (spatial-based working memory) of the previous choice (Paul *et al.*, 2009).

Interestingly (Fig. 4C), in line with previous literature findings (King and Arendash, 2002; Deacon *et al.*, 2008; Yassine *et al.*, 2013), the spontaneous alternation task did not reliably detect cognitive deficits in Tg2576 mice at 6 months of age because no difference was found in their working-memory performance in comparison to cognitively intact, WTs, both in spontaneous alternation and total entries into the arms (spontaneous alternation one-way ANOVA *F*(2,12) = 0.15 *P* = 0.86; Fisher’s *post hoc* test WT versus Tg2576 *P* = 0.99; Tg2576 versus Tg2576 + mAb *P* = 0.68; Total Entries *F*(2,12) = 0.28 *P* = 0.76; Fisher’s *post hoc* test WT versus Tg2576 *P* = 0.81; Tg2576 versus Tg2576 + mAb *P* = 0.72). All three groups of mice alternated between arms above chance level (22.2%), indicating that neither cohort showed impairment in this test. On the other hand (Fig. 5C) and in line with previous reports (Spires-Jones and Knafo, 2012; Ameen-Ali *et al.*, 2017), although disability was clearly discernible in *naïve*, cognitively impaired 3xTg at 6 months of age when tested in comparison to age-matched WTs (Spontaneous alternation one-way ANOVA *F*(2,28) = 7.44 *P* = 0.025; Total entries *F*(2,28) = 18.01 *P* = 0.00001), no significant improvement in their reference and working-memory/learning abilities was detected following systemic injection with 12A12mAb (Fisher’s *post hoc* test analysis, Total entries: WT versus 3xTg ***P < 0.0005; WT versus 3xTg + mAb ***P < 0.0005; 3xTg versus 3xTg + mAb *P* = 0.19; Spontaneous alternation WT versus 3xTg ***P < 0.0005; WT versus 3xTg + mAb *P < 0.05; 3xTg versus 3xTg + mAb *P* = 0.17). In this framework, it is worth stressing that, in contrast to 3xTg characterized by genetically driven tau pathology, Tg2576 mice express human APP (K670N/M671L)PS1(M146V) transgene in endogenous background of murine not-mutated tau. Therefore, the discrepancy in results between two different Tg Alzheimer’s disease rodent models, each having its own characteristics, may be due to the aggressive phenotype of the human tau-overexpressing 3xTg strain, which is likely to require a more optimized immunization regimen (antibody dosage, time of treatment, timing of administration) in order to fully prevent and/or delay its robust cognition symptomatology. Alternatively, the reversible nature of *in vivo* tau neuropathology could be restricted within strain-specific temporal window(s) because of the complex and multifactorial feature of Alzheimer’s disease pathology involving a wide range of inflammatory, oxidative, neurodegenerative causative mechanisms (Webster *et al.*, 2014; Velazquez *et al.*, 2018).

Finally, to rule out the possibility that the *in vivo* enhancement of cognitive skills in immunized animals involved an effect of 12A12mAb treatment on body energy homeostasis known to influence their sensorial-motor abilities, metabolic rate (EE) from vehicle- or antibody-infused non-Tg WT mice was assessed by means of indirect calorimetry during 2 days of continuous analysis/recording. As shown in Supplementary Fig. 2A, an unpaired *t*-test of EE data revealed no significant difference between the two experimental groups [vehicle-treated animals: (*M* = 25.29; SEM = ±0.61) or mAb-treated animals: (*M* = 24.92; SEM = ±0.64 *t*_(142)_ = 0.42)]. Furthermore, the EE analysis in resting conditions (REE)—i.e. by considering only the EE values generated in the absence of motor activity (i.e. 0–2 counts)—did not show any variation in heat production from 12A12mAb-treated healthy WT mice, thus corroborating the important finding that intra-caudal injection either with vehicle alone (*M* = 18.51; SEM = ±0.43), or with antibody (*M* = 18.08; SEM = ±0.52 *t*_(142)_ = 0.42), was ineffective in altering the whole body REE, whatever the physical motor activity involved (*t*_(118)_ = 0.63) (Supplementary Fig. 2B).

In keeping with this finding, no change in recognition memory performance was detected when vehicle- or 12A12mAb-treated WT mice were evaluated in the NOR paradigm (Supplementary Fig. 2C and D), further indicating that the immunization regimen *per se* did not affect cognitive functions under non-pathological settings. Two-way ANOVA of time (s) of exploration of FO versus NO showed no significant difference for treatment factor (*F*(1,12) = 0.28 *P* = ns), significant object novelty factor (*F*(1,12) = 18.74 *P* < 0.001) and no significant effect of the interaction between treatment and object novelty (*F*(1,12) = 0.08 *P* = ns). *Post hoc* Tukey’s test for object novelty (FO versus NO) further confirmed that both vehicle-treated (**P < 0.01) and mAb-treated (**P < 0.01) WT animals exhibited intact recognition memory (Supplementary Fig. 2C). No difference was found between the PI of vehicle-treated and mAb-treated WT groups (unpaired sample *t*-test: vehicle-treated and mAb-treated ones, *t*_(6)_ = 1.672, *P* = ns, Supplementary Fig. 2D), thus demonstrating that 12A12mAb-induced injection did not impair recognition memory in non-Tg mice. In agreement with the cleavage-selectivity of antibody (Supplementary Fig. 1), the 12A12mAb treatment appeared to be avoid of potential adverse side-effects in discriminatory skills when injected in healthy animals, notwithstanding its ability of penetrating the animals’ blood–brain barrier and/or successfully accessing to hippocampus in biologically active state (Fig. 1D).

Likewise, no difference in cognitive performance was detected when sham-immunized 6-month-old Tg2576 mice (i.e., animals administered with IgG isotype control, at the same dosage and period of time) were tested for performance in NOR paradigm in comparison with their vehicle-treated counterparts. Two-way ANOVA analysis on time (s) of exploration of FO versus NO displayed no significant difference for object factor (*F*(1,8) = 0.66 *P* = ns) and treatment factor (*F*(1,8) = 0.67 *P* = ns) in vehicle- and IgG-treated Tg mice (Supplementary Fig. 3A). Moreover, the unpaired sample *t*-test of PI data showed that neither vehicle nor IgG administration improved the deficit of recognition memory (compare Supplementary Fig. 3B with Supplementary Fig.2C), thus confirming the lack of ability of Tg animals in discriminating between FO and NO (*t*_(4)_ = 0.05, *P* = ns). Similar results were detected following IgG infusion in 6-month-old 3xTg mice when compared to not-immunized Tg counterparts (data not shown).

Active behaviour, such as exploring a novel environment, induces the expression of the immediate-early gene Arc (activity-regulated cytoskeletal-associated protein, or Arc/Arg3.1) in several brain regions, including the hippocampus. Activity-regulated cytoskeleton-associated protein messenger RNA is quickly induced and dynamically up-regulated by behavioural experience and the protein is selectively translated into activated dendrites, being required for the memory consolidation of an early initial potentiation of synaptic transmission into a lasting form of long-term potentiation (LTP) (Ramirez-Amaya *et al.*, 2005 , 2013; Plath *et al.*, 2006; Korb and Finkbeiner, 2011). Interestingly and consistent with their cognitive enhancement in behavioural assessments (Figs 4 and 5), western blotting analyses performed on hippocampal synaptosomal-enriched preparations isolated from post-trained animals (Supplementary Fig. 4A and B) showed that the stimulus-driven, steady-state expression level of activity-regulated cytoskeleton-associated protein was significantly up-regulated in 12A12mAb-immunized Tg2576 and 3xTg mice when compared to their saline-treated cognitively impaired counterparts (one-way ANOVA followed by Bonferroni’s *post hoc* test *F*(2,18) = 34.81 *P* < 0.0001 Tg2576; *F*(2,18) = 33.32 *P* < 0.0001 3xTg; Tg2576 + mAb versus Tg2576 ****P < 0.0001; Tg2576 + mAb versus WT n.s. *P* = 0.1441; 3xTg + mAb versus 3xTg ***P < 0.0005; 3xTg + mAb versus WT **P < 0.01). Conversely and in line with their scarce performance in novelty-based cognitive tasks (Figs 4 and 5), *naïve* Alzheimer’s disease Tg animals—which were not systemically infused with 12A12mAb—displayed marked defects in the experience-dependent induction of activity-regulated cytoskeleton-associated protein expression, and then in their processes of memory/learning trace consolidation following its initial acquisition, as shown by the finding that immunoreactivity signal of protein in their synaptic fractions was significantly lower than that from healthy WT controls (one-way ANOVA followed by Bonferroni’s *post hoc* test ****P < 0.0001 for Tg2576 versus WT and for 3xTg versus WT).

Collectively, these experiments indicate that passive immunization with 12A12mAb, which selectively targets the neurotoxic NH_2_htau fragment(s) *in vivo*, significantly improves cognition in symptomatic (6 months old) Tg-Alzheimer’s disease animals by rescuing their instinctual and innate preference for novelty (object recognition and object location skills) when tested in two pathologically relevant, hippocampal-dependent behavioural tasks.

### Loss in dendritic spine density is prevented in hippocampal CA1 region from 12A12mAb-infused 6-month-old Tg-Alzheimer’s disease animals

**Figure 6:**
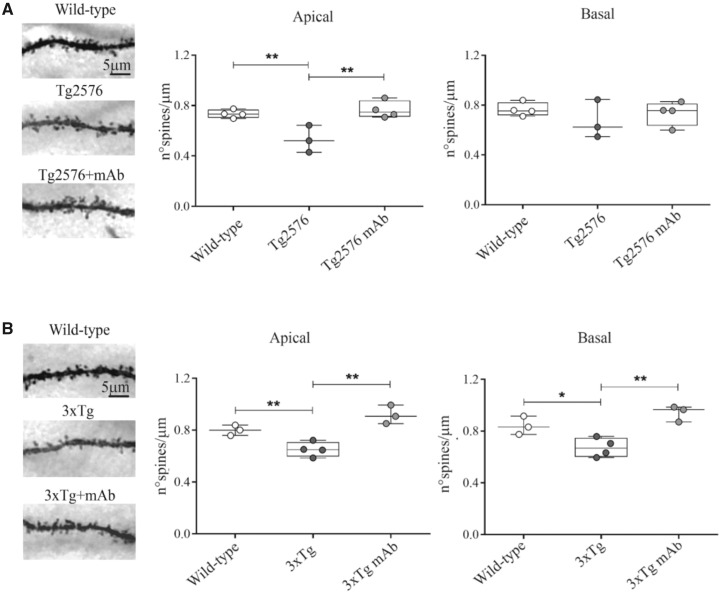
**Immunization with 12A12mAb in Tg-Alzheimer’s disease mice is protective against the dendritic spines density loss which affects the memory and learning processes.** (**A**, **B**) Comparative photomicrographs of Golgi-stained hippocampal CA1 neurons showing dendritic segments from animals from three experimental groups (WT, Tg-Alzheimer’s disease and Tg-Alzheimer’s disease + mAb) of both strains (Tg2576, 3xTg) (left, refers to CA1 pyramidal neurons dendrites scale bar: 5 µm). Box-and-whisker plots (right) depict the morphometric analysis of the dendritic spine density from the three experimental groups. Values are expressed as number of spines per 1 µm segment. Statistically significant differences (comparisons were made on single mouse values obtained by averaging the number of spines counted on neurons of the same mouse) were calculated by ANOVA followed by *post hoc* test for multiple comparison among more than two groups. *P* < 0.05 was accepted as statistically significant (*P < 0.05; **P < 0.01; ***P < 0.0005; ****P < 0.0001).

Dendritic spines, the sites of excitatory synapses protruding from dendritic shafts, are cellular morphological specializations devoted to memory-forming processes in neurons (Segal, 2005). Being extremely dynamic structures, modification in their number, size and/or shape is an important index of synaptic plasticity occurring in response to external environmental inputs (Pignataro *et al.*, 2015). As a consequence, loss of dendritic arbourization (length/complexity) in vulnerable neuronal networks, although occurring along different spatio-temporal patterns among commonly used Tg animal models, undoubtedly represents one of the earliest changes of structural plasticity which critically contributes over time to the disruption of neuronal network with consequent appearance of cognitive dysfunction in Alzheimer’s disease and other related dementias (Knobloch and Mansuy, 2008; Spires-Jones and Knafo, 2012; Cochran *et al.*, 2014; Dorostkar *et al.*, 2015). Therefore, in order to complement our behavioural findings, we assessed the neuroanatomical effect of passive immunization with 12A12mAb on dendritic connectivity from 6-month-old Alzheimer’s disease animals. To this aim, hippocampal sections from mice of the three experimental groups were stained by Golgi-Cox impregnation procedure followed by quantitative assessment of dendritic spine density (number of spines per unit length) along both apical and basal compartments of individual CA1 pyramidal neurons (Fig. 6).

As shown in Fig. 6A and in line with previous works reporting in Tg2576 an early decline of dendritic boutons which undergo dystrophy and shrinkage (Lanz *et al.*, 2003; Jacobsen *et al.*, 2006; D’Amelio *et al.*, 2011), the spine loss was detectable at the age of 6 months in apical compartments of CA1 hippocampal neurons from this genetic background when animals were compared to non-Tg controls. Importantly, in 12A12mAb-immunized Alzheimer’s disease group the apical spine density was significantly ameliorated up to the level of saline-injected cognitively intact WTs (one-way ANOVA followed by Fisher’s *post hoc* test *F*(2,8) = 10.828, *P* = 0.00530; **P < 0.01 WT versus Tg2576; **P < 0.01 Tg2576 + mAb versus Tg2576), indicating that treatment was strongly effective in blocking/preventing the dendritic degeneration. Interestingly, no difference was detected when spines were counted in the basal compartment of CA1 neurons from the three experimental cohorts (one-way ANOVA *F*(2,8) = 0.71926, *P* = 0.51611), suggesting that age-related spine changes in Tg2576 mice initially involve the apical dendritic arbours with no apparent effect on basal dendrites of CA1 pyramidal neurons which are more likely to be affected only later, when their structural plasticity and stability (formation and elimination) is completely impaired (Spires-Jones *et al.*, 2007).

On the other hand and in stark contrast with previous literature findings (BIttner *et al.*, 2010), we found out (Fig. 6B) that the reduction in the spines density, both in apical and basal compartments of individual CA1 pyramidal neurons, already started from the age of 6 months in cognitively impaired 3xTg mice (one-way ANOVA followed by Fisher’s *post hoc* test apical: *F*(2,7) = 18.697, *P* = 0.00156; basal: *F*(2,7) = 13.404, *P* = 0.00404) which exhibited lower values in dendritic protrusions counts when compared with their age-matched, non-Tg WTs. Remarkably, degeneration of dendritic spine structures was robustly decreased in immunized 3xTg mice (apical: **P < 0.01 3xTg + mAb versus 3xTg; **P < 0.01 WT versus 3xTg; basal: **P < 0.01 3xTg + mAb versus 3xTg; *P < 0.05 WT versus 3xTg) pointing out that—possibly as result of increased afferent inputs to the CA1 from other neighbouring hippocampal areas and/or as a local positive effect in the CA1 region—12A12mAb treatment was able to mitigate the age-related pathology of post-synaptic connections from symptomatic 6-month-old 3xTg mice, both in their apical and basal compartments.

### Systemic administration of 12A12mAb also normalizes the Alzheimer’s disease-related electrophysiological alterations of Tg-Alzheimer’s disease animal models

**Figure 7:**
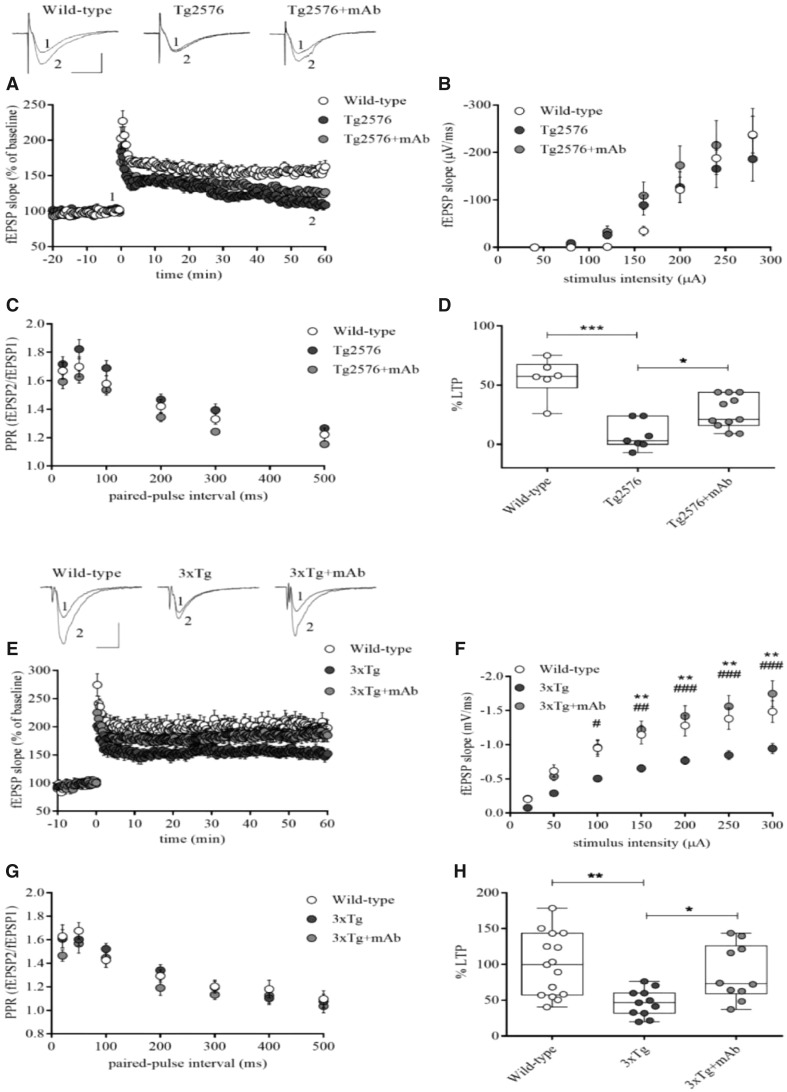
**Reduction of cognitive deficits in 12A12mAb-immunized Tg-Alzheimer’s disease mice correlates with an increased LTP.** (**A**, **D**) For Tg2576; (**E**, **H**) for 3xTg) time plot of average fEPSP responses (**A**, **E**) and changes in magnitude of LTP at CA3-Ca1 synapses (**D**, **H**) were calculated among animals (*n* = 6–10) from three experimental groups (WT, Tg-Alzheimer’s disease and Tg-Alzheimer’s disease + mAb) of both strains. At least seven slices from six different mice were recorded for each experimental condition. Data are presented as the mean (±SEM). The traces above the plot show fEPSPs at baseline (1) and at 60 min after LTP induction (2). The box-whisker plots show pooled data. Statistically significant differences (see details in the main text) were calculated by ANOVA followed by *post hoc* test for multiple comparisons among more than two groups. *P* < 0.05 was accepted as statistically significant (*P < 0.05; **P < 0.01; ***P < 0.0005; **** *P* < 0.0001). (**B**, **C**) For Tg2576; (**F**, **G**) for 3xTg) input/output curves show the fEPSP slopes plotted against the corresponding stimulus intensities recorded from hippocampal slices of animals (*n* = 6–10) from three experimental groups (WT, Tg-Alzheimer’s disease and Tg-Alzheimer’s disease + mAb) of both strains (**B**, **F**). Comparison of PPF in animals (*n* = 6–10) from three experimental groups (WT, Tg-Alzheimer’s disease and Tg-Alzheimer’s disease + mAb) of both strains (Tg2576, 3xTg) was also determined (**C**, **G**). PPF was induced by pairs of stimuli delivered at increasing interpulse intervals (20, 50, 100, 200, 300, 500 ms). Data are presented as the mean (±SEM) facilitation ratio of the second response relative to the first response. Statistically significant differences (see details in the main text) were calculated by ANOVA followed by *post hoc* test for multiple comparisons among more than two groups. *P* < 0.05 was accepted as statistically significant (*P < 0.05; **P < 0.01; ***P < 0.0005; ****P < 0.0001). fEPSP = field excitatory post-synaptic potential; PPF = paired-pulse facilitation.

In order to investigate whether 12A12mAb immunization, in addition to its protective actions on Alzheimer’s disease-related behavioural and neurochemical and neuroanatomical abnormalities, was also able to exert an effect on electrophysiological correlate(s) of the memory/learning processes, hippocampal synaptic transmission and plasticity in the Schaffer collateral pathway were compared between Tg and WT animals from both genetic backgrounds (Fig. 7A–D for Tg2576; Fig. 7E–H for 3xTg).

We first recorded basal synaptic transmission and the strength of pre-synaptic Schaffer collaterals activation (i.e. axonal depolarization) from CA3-to-CA1 synapses in acute brain slices from 6-month-old WT and age-matched Tg2576 animals treated with saline vehicle or 12A12mAb, respectively. To this aim, we first generated input/output curves by measuring the field excitatory post-synaptic potentials elicited in the stratum radiatum of the CA1 area after stimulation of the Schaffer collaterals at increasing intensities. As shown in Fig. 7A and B and in line with previous investigations reporting no change in basal synaptic transmission in this Tg Alzheimer’s disease model at 6 months of age (Chapman *et al.*, 1999; Nobili *et al.*, 2017), the input/output curves displayed a similar trend among the three experimental groups (two-way repeated-measures ANOVA for stimulus intensity × experimental group followed by Bonferroni’s *post hoc* test *F*(12,282) = 0.8409 *P* = 0.6082; n.s. *P* > 0.05 for all comparisons).

Next, we investigated the pre-synaptic function by assessing paired-pulse facilitation, a short-term plasticity paradigm which inversely depends on Ca^2+^-dependent pre-synaptic changes in neurotransmitter release probability at nerve endings (Manabe *et al.*, 1993; Debanne *et al.*, 1996; Dobrunz and Stevens, 1997; Dobrunz *et al.*, 1997; Thomson, 2000; Zucker and Regehr, 2002). Again (Fig. 7C), short-term potentiation was almost identical among the three animals’ cohorts (two-way repeated-measures ANOVA for paired-pulse interval × experimental group, followed by Bonferroni’s *post hoc* test *F*(10,170) = 0.51 *P* = 0.8839; n.s. *P* > 0.05 for all comparisons), consistent with previous results referring no significant dissimilarity in paired-pulse facilitation between 6-month-old Tg2576 and WT littermates (Jung *et al.*, 2011; Nobili *et al.*, 2017).

In contrast to the basic synaptic transmission (input–output relationship and paired-pulse facilitation), the ‘classical’ *n*-methyl-d-aspartate (NMDA) receptor-dependent LTP paradigm at Schaffer collaterals/CA1 synapses—a long-lasting enhancement of the strength/efficacy of excitatory synaptic transmission which is widely used in investigations on numerous APP/Aβ models of Alzheimer’s disease (Rowan *et al.*, 2003; Shankar *et al.*, 2008)—turned out to be significantly compromised in 6-month-old Tg2576 mice in comparison to age-matched WTs (Fig. 7D). Following the induction of LTP by delivery of trains of high-frequency stimulation at half-maximal intensity, field excitatory post-synaptic potential slopes appeared to decay down to baseline in 6-month-old Tg2576 animals so that no persistent, activity-driven potentiation was measurable starting from 30 min after induction which was indicative of an impaired function of hippocampal Schaffer collaterals/CA1 synapses. Importantly and in keeping with improvement of cognitive performance in hippocampal-dependent behavioural assessments, peripheral administration of 12A12mAb was able to mitigate *in vivo* the disease-related LTP deficiency of symptomatic Tg2576 animals, as shown by the fact that the LTP amplitude calculated after application of high-frequency stimulation was significantly increased in immunized experimental group when compared to ‘naïve’ cognitively impaired counterpart (one-way ANOVA followed by Bonferroni’s *post hoc* test *F*(2,21) = 19.38 *P* < 0.0001; ***P < 0.0005 Tg2576 versus WT; *P < 0.05 Tg2576 + mAb versus Tg2576; Tg2576 + mAb versus WT **P < 0.01). Moreover, these electrophysiological investigations further corroborated the finding that the disruption of synaptic plasticity in hippocampal Schaffer collateral commissural pathway from this Alzheimer’s disease model was more likely due to altered post-synaptic signalling pathways, given that no alteration in paired-pulse facilitation was contextually detected in Tg animals at 6 months of age (Chapman *et al.*, 1999; Jacobsen *et al.*, 2006; Jung *et al.*, 2011; Nobili *et al.*, 2017).

In contrast with results from symptomatic Tg2576 mice, in 3xTg paradigm the input/output relationship revealed a significant reduction of field excitatory post-synaptic potential slopes evoked by increasing stimulation intensities (Fig. 7E and F) when 6-month-old Tg animals were compared to WT counterparts (two-way repeated-measures ANOVA for stimulus intensity × experimental group followed by Bonferroni’s *post hoc* test *F*(12,204) = 5.812 *P* < 0.0001; *P < 0.05 and **P < 0.01 WT versus 3xTg for paired comparisons). Most importantly, cumulative distributions of field excitatory post-synaptic potential slopes within the range of 100 and 300 μA of stimulus amplitude were shifted to higher values in 12A12mAb-immunized Alzheimer’s disease group in contrast to ‘naïve’, cognitively impaired counterpart, indicating that antibody treatment positively influenced the fast glutamatergic transmission in this genetic background (^#^*P* < 0.05, ^##^*P* < 0.01, ^###^*P* < 0.001 for 3xTg versus 3xTg + mAb for paired comparisons with Bonferroni’s *post hoc* test). No change in paired-pulse facilitation short-term plasticity (Fig. 7G) was found among the three experimental cohorts (two-way ANOVA paired-pulse interval × genotype followed by Bonferroni’s *post hoc* test *F*(12,198) = 0.3464 *P* = 0.9792 n.s. *P* > 0.05 for all comparisons), consistent with previous data showing that the abnormalities in pre-synaptic release machinery were not discernible between 6-month-old 3xTg and age-matched WTs when measured in a facilitation electrophysiological paradigm (Oddo *et al.*, 2003). In a way similar to Tg2576, hippocampal slices from 6-month-old 12A12mAb-injected 3xTg animals displayed a strong potentiation after high-frequency stimulation bout, pointing to a strong protective action evoked *in vivo* by the antibody treatment on the cellular/molecular correlate(s) of their memory/learning processes (one-way ANOVA followed by Bonferroni’s *post hoc* test *F*(2,33) = 7.018 *P* = 0.0029; **P < 0.01 3xTg versus WT; *P < 0.05 3xTg + mAb versus 3xTg; 3xTg + mAb versus WT n.s. *P* > 0.05). Notably, when LTP was calculated in 6-month-old 3xTg mice (Fig. 7H), a lower post-tetanic potentiation was found against WTs suggesting that, in this Alzheimer’s disease strain, the LTP reduction in magnitude and persistence was more likely due to deficits of induction (either pre- and/or post-synaptic), in line with structural and functional modifications observed both in their basal synaptic transmission and dendritic spine density (Fig. 6B).

Taken together, these electrophysiological recordings indicate that disruption of excitatory synaptic transmission and plasticity detected at 6 months of age in hippocampal CA3-CA1 circuit of these two genetically distinct Tg-Alzheimer’s disease animal models, although manifests at different rate and involves non-overlapping causative mechanism(s), was significantly rescued following *in vivo* peripheral administration of 12A12mAb.

### Expression levels of inflammatory astroglial and microglial markers are also down-regulated in 6-month-old 12A12mAb-immunized Tg-Alzheimer’s disease animals

The inflammatory response—which is one of the earliest manifestations of neurodegenerative tauopathies, including Alzheimer’s disease (Bellucci *et al.*, 2004; Yoshiyama *et al.*, 2007; Wes *et al.*, 2014; Leyns *et al.*, 2017; Ishikawa *et al.*, 2018)—may act as a double-edged sword being either detrimental or protective depending on the context (Schlachetzki and Hull, 2009). On the one hand, activated glial cells contribute to the Alzheimer’s disease pathogenesis by means of diverse mechanisms including complement-mediated synapse removal, non-cell autonomous spreading of pathological seeds/conformers, extracellular release of inflammatory mediators such as pro-inflammatory cytokines, complement components, chemokines, free radicals and gliotransmitters which, in turn, trigger neurodegeneration. On the other hand, astro- and microglial reactions are endowed with beneficial role in Alzheimer’s disease environment by stimulating the digestion/clearance of pathogenic Aβ and tau species and, then, by preventing their accumulation into insoluble cerebral lesions, the senile plaques and neurofibrillary tangles.

**Figure 8:**
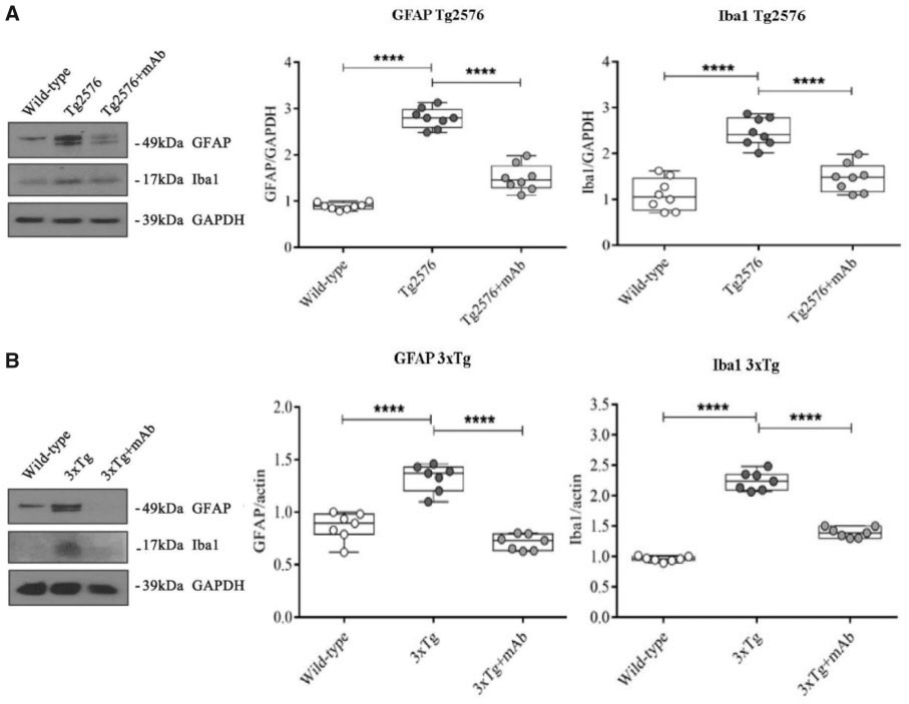
**Inflammatory response (activation of astrocytes and microglia) is strongly down-regulated in 12A12mAb-immunized Tg-Alzheimer’s disease mice.** (**A**, **B**) Neuroinflammation processes (activation of astrocytes and microglia) were assessed on hippocampal extracts from animals from three experimental groups (WT, Tg-Alzheimer’s disease and Tg-Alzheimer’s disease + mAb) of both strains (Tg2576, 3xTg) by western blotting analysis (left) for inflammatory proteins (glial fibrillary acidic protein, Iba1). Relative densitometric quantification of intensity signals (right) indicates lower levels of glial fibrillary acidic protein and Iba1 in Tg-Alzheimer’s disease mice + mAb compared to not-immunized Tg-Alzheimer’s disease. GAPDH housekeeping expression serves as loading control. Arrows on the right side indicate the molecular weight (kDa) of bands calculated from migration of standard proteins. Full uncropped blots are available in Supplementary Fig. 8. Values are from at least three independent experiments and statistically significant differences (see details in the main text) were calculated by ANOVA followed by *post hoc* test for multiple comparisons among more than two groups. *P* < 0.05 was accepted as statistically significant (*P < 0.05; **P < 0.01; ***P < 0.0005; ****P < 0.0001). GAPDH = glyceraldehyde-3-phosphate dehydrogenase; GFAP = glial fibrillary acidic protein.

To get further insights into protective effect evoked by i.v. 12A12mAb-based immunization in Tg-Alzheimer’s disease mice, the extent of inflammatory response was assessed on hippocampi from 6-month-old Tg2576 and 3xTg mice of the three experimental groups (WT, ‘naïve’ Tg-Alzheimer’s disease, Tg-Alzheimer’s disease + mAb). Western blotting analysis (Fig. 8) were carried out on animals’ total extracts by probing with antibodies which detect the glial fibrillary acidic protein and Iba1, whose cell type-specific steady-state expression levels are recognized as indicative of active astrogliosis and microgliosis, respectively (Sydow *et al.*, 2016). As shown in Fig. 8A and B and regardless of the different genetic background, the immunoreactivity signals of these two classical inflammatory markers were strongly increased in saline-treated, ‘naïve’ Tg-Alzheimer’s disease mice in comparison to WT controls, in line with previous findings reporting a prominent astrocytic and microglial activation in hippocampal parenchyma from these animal models (Olabarria *et al.*, 2010, 2011; Vogels *et al.*, 2019). Remarkably, the gliosis detected in 12A12mAb-injected Tg-Alzheimer’s disease mice turned out to be significantly down-regulated compared to their *naïve* counterparts (one-way ANOVA followed by Bonferroni’s *post hoc* test glial fibrillary acidic protein: *F*(2,21) = 169.4 *P* < 0.0001 Tg2576 ****P < 0.0001 for all comparisons; *F*(2,18) = 53.88 *P* < 0.0001 3xTg; ****P < 0.0001 3xTg versus WT; ****P < 0.0001 3xTg + mAb versus 3xTg; n.s. *P* > 0.05 3xTg + mAb versus WT; Iba1: *F*(2,21) = 38.43 *P* < 0.0001 Tg2576 ****P < 0.0001 Tg2576 versus WT; ****P < 0.0001 Tg2576 + mAb versus Tg2576; n.s. *P* > 0.05 Tg2576 + mAb versus WT; *F*(2,18) = 273 *P* < 0.0001 3xTg ****P < 0.0001 for all comparisons), indicating that: (i) the neuron-derived, extracellularly released 20–22 kDa NH_2_htau form(s) is more likely to be endowed with non-cell autonomous action by contributing to the glial cells activation; (ii) the neuroprotective effect of 12A12mAb appears to be, at least in part, due to its modulatory role at the glia-neurons interplay.

Taken together these findings indicate that sub-chronic i.v. delivery of 12A12mAb into the hippocampus is devoid of potentially adverse inflammatory effects associated to classical immunization regimen by limiting the local activation of neuroglia which is *per se* both a consequence to the disease process and a contributor to the synaptic pathology and neuronal damage (Block *et al.*, 2007; Yoshiyama *et al.*, 2007; Edison *et al.*, 2008; Perry *et al.*, 2010; Schwab *et al.*, 2010; Zotova *et al.*, 2010).

## Discussion

Accumulating evidence have suggested that the detrimental effects of Aβ are dependent on tau pathology (Rapoport *et al.*, 2002; King *et al.*, 2006; Roberson *et al.*, 2007; Ittner *et al.*, 2010; Vossel *et al.*, 2010; Desikan *et al.*, 2011; Shipton *et al.*, 2011; Nussbaum *et al.*, 2012; Bloom, 2014) and that tau, rather than Aβ (Murray *et al.*, 2015), serves a prominent role in early synaptic decline and cognitive impairment (Arriagada *et al.*, 1992; Ashe and Zahs, 2010; Nelson *et al.*, 2012; Pontecorvo *et al.*, 2017; Busche *et al.*, 2019). Independently of its ability of seeding aggregation, abnormal extracellular/intracellular tau is *per se* neurotoxic (Diaz-Hernandez *et al.*, 2010; Medina and Avila, 2014*a*, *b*; Hu *et al.*, 2018) and propagates trans-synaptically along interconnected neuronal networks in a stereotypical manner which strongly correlates with the development of clinical symptoms during Alzheimer’s disease progression (Mohamed *et al.*, 2013; Pooler *et al.*, 2013; Yamada *et al.*, 2017). These pathologically relevant findings represent the rationale which advocates the employment of tau-based strategies (Li and Gotz, 2017; Congdon and Sigurdsson, 2018) as promising disease-modifying intervention of slow progressing Alzheimer’s disease and other human dementias (Novak *et al.*, 2018*a*; Jadhav *et al.*, 2019), especially in view of the disappointing outcomes from Aβ-targeting pharmacological and immunological approaches (Sigurdsson, 2008; Giacobini and Gold, 2013; Doody *et al.*, 2014; Salloway *et al.*, 2014; Schroeder *et al.*, 2016; Agadjanyan *et al.*, 2017). In this connection, tau-directed passive immunotherapy—which relies on the specific, epitope-directed antibody-mediated depletion/clearance of its toxic species (Sigurdsson, 2008; Pedersen and Sigurdsson, 2015; Li and Gotz, 2017; Congdon and Sigurdsson, 2018)—has been recently recognized as a feasible, valuable approach to reduce the neuropathology and to improve the memory/learning abilities of experimental animal models of tauopathies (Boutajangout *et al.*, 2011; Chai *et al.*, 2011; d’Abramo *et al.*, 2013; Yanamandra *et al.*, 2013, 2015; Castillo-Carranza *et al.*, 2014; Dai *et al.*, 2015, 2018; Subramanian *et al.*, 2017). However, several reasons have hindered the clinical success of tau-targeting approaches which are currently under investigation (Giacobini and Gold, 2013; Novak *et al.*, 2018a; Elmaleh *et al.*, 2019). To this regard, recent reports of ongoing trials indicate that the potential flaws include: (i) study design with the medical care given too late when neuronal damage is already present in a considerable extent so that drugs/modulators are unable to compensate adequately for the detrimental effects; (ii) systemic toxicity owing to long-term and multiple administrations of drugs/modulators used at high doses which can interfere with the neuronal physiology (i.e. affect the normal cellular metabolism and/or impact on the immune surveillance); (iii) inadequate brain bioavailability of medicaments against the target substrate and/or the biochemical pathway, even after crossing the blood–brain barrier; (iv) adverse risks of inflammatory response, such as cerebral microhaemorrhages (Elmaleh *et al.*, 2019).

Here, we show that systemic administration with 12A12mAb—which selectively recognizes the human tau at D_25_(DRKD_26_QGGYTMHQDQEGDTDAGLK_44_), a known N-terminal truncation protein site (Quinn *et al.*, 2018) previously identified both in cellular and animal Alzheimer’s disease paradigms (Corsetti *et al.*, 2008) and in human Alzheimer’s disease brains (Rohn *et al.*, 2002; Amadoro *et al.*, 2012; Corsetti *et al.*, 2015)—rescues the neurochemical, anatomical, behavioural and electrophysiological alterations underlying the Alzheimer’s disease phenotype in two well-established Tg mouse strains, such as preclinical Tg2576 and 3xTg models. Of particular relevance is the fact that the Tg Tg2576 mice expressing human mutant APP (K670N/M671L), in contrast to 3xTg harbouring PS1(M146V), APP(Swe) and tau(P301L) transgenes, display an endogenous genetic background of murine not-mutated tau. Furthermore, since treatments started when synaptic deterioration is evident but extensive neurodegeneration has not yet developed turn out to be the most effective in preventing the disease-associated brain atrophy and related cognitive impairment (Bokde *et al.*, 2009; Elmaleh *et al.*, 2019), our experimental evaluations are carried out on symptomatic animals which are employed at early-middle stages (6 months old) of pathology progression, when their hippocampi are not largely compromised with massive neuronal loss. It is also worth underlining that the 12A12mAb we employed in the present study is specific for the pathological truncated tau because it selectively binds *in vivo* the neurotoxic Alzheimer’s disease-linked NH_2_26-230 fragment (i.e. NH_2_htau) without showing any significant cross-reaction towards the intact, physiological form of protein, in line with our previous investigations (Amadoro *et al.*, 2012). To this point, biochemical and functional outcomes *in vivo* measures further confirm that 12A12mAb: (i) does not specifically interact with the abundant intracellular pool of endogenous normal full-length tau protein whose steady-state level is unchanged in hippocampus after its i.v. delivery in Tg-Alzheimer’s disease mice regardless of the genetic backgrounds; (ii) is harmless when injected in healthy, cognitive-intact WT mice, despite the ability of successfully penetrating/reaching the brain in its biologically active (antigen-competent) state under physiological settings. Remarkably, the cleavage-specific 12A12mAb—which selectively binds 20–22 kDa NH_2_htau without unproductive and deleterious cross-reaction towards the physiological intact tau—appears to be potent tool by providing measurable changes on Alzheimer’s disease brain physiopathology which result in significant improvement of the synaptic and cognitive deficits in affected animals, even after its short-term (14 days) i.v. delivery. Conversely, there is proof that the use of other therapeutic anti-tau antibodies binding all forms of tau is more likely to result in considerable reduction of its effective dose available *in vivo* against the target toxic tau species with consequent requirement of more aggressive and prolonged applications (Novak *et al.*, 2018*a*). Furthermore, our results may have important clinical implications by prospecting the non-invasive i.v. delivery route of 12A12mAb as effective and safe disease-modifying approach in contrasting the earliest neuropathological and cognitive alterations of subjects which suffer the chronically developing human Alzheimer’s disease and non-Alzheimer’s disease tauopathies characterized by an increased burden of tau truncation. In post-mitotic neurons, tau is endowed with important functions beyond the control of microtubule integrity and dynamics (Sotiropoulos *et al.*, 2017) and the treatment with tau-targeting antibodies may have undesirable adverse side-effects due to ‘loss of function’ of full-length protein (Rosenmann *et al.*, 2006; Rozenstein-Tsalkovich *et al.*, 2013; Bakota *et al.*, 2017). Although we cannot rule out the later development of gliosis following prolonged immunization regimen, from a translational perspective another interesting finding of the present study is that the sub-chronic i.v. treatment with 12A12mAb is sufficient *per se* to drive a robust therapeutic effect in the absence of increased microglia and astrocyte activation which, on the contrary, appears to be critical for the mechanism of action of at least a few Aβ-directed antibodies (Bard *et al.*, 2000; Wilcock *et al.*, 2004) leading as byproduct to excessive deleterious stimulation of local inflammatory response (Lemere, 2013; Wisniewski and Goni, 2015). We find no obvious evidence of neuroinflammatory response which is known to cause mortality in WT mice when actively immunized with various fragments of tau (Rosenmann *et al.*, 2006; Rozenstein-Tsalkovich *et al.*, 2013). Furthermore, the evidence that passive immunization with 12A12mAb can normalize *in vivo* the APP/Aβ dysmetabolism in two independent genetic backgrounds overexpressing human mutated APP (K670N/M671L) not only unveils a novel and potential connection between tau and APP/Aβ, whereby toxic tau can upstream affect APP/Aβ pathology in damaging synapses, but also—and more importantly—highlights the 20–22 kDa NH_2_-terminal tau fragment as crucial target for Alzheimer’s disease therapy starting from its earliest stages which are characterized by initial disruption of synaptic functions in the absence of frank neuronal loss. Therefore, this study prospects the peripheral administration of the humanized counterpart of murine 12A12mAb as a novel, promising multi-targeted intervention in preventing disease-associated cognitive deterioration in human beings suffering Alzheimer’s disease-related dementias, being endowed with higher clinical potentialities than those altering either neuropathology alone (Oddo *et al.*, 2006; Rosenmann *et al.*, 2006; Lambracht-Washington and Rosenberg, 2013; Bakota *et al.*, 2017).

Concerning the mechanism(s) of action involved in the beneficial power of 12A12mAb immunization, in the present study, we are unable to anticipate whether tau-directed therapeutic effects offered by i.v. delivery of 12A12mAb involve only the extracellular or both intracellular and extracellular pool of toxic truncated tau because we did not collect and analyse the level of NH_2_htau fragment in CSF or interstitial fluid and plasma. It is worth noting that the N-terminal, but not C-terminal, fragments of tau including the 20–22 kDa NH_2_htau form(s), are mainly secreted from synaptosomes of Alzheimer’s disease brains (Sokolow *et al.*, 2015) and detected both in CSF from Alzheimer’s disease patients (Johnson *et al.*, 1997; Portelius *et al.*, 2008; Meredith *et al.*, 2013; Amadoro *et al.*, 2014; Chen *et al.*, 2019; Cicognola *et al.*, 2019) and in conditioned media from patient-derived induced pluripotent stem cells cortical neurons (Bright *et al.*, 2015; Kanmert *et al.*, 2015; Sato *et al.*, 2018). The NH_2_26-44 amino acidic stretch, which is the minimal biological active moiety of parental 20–22 kDa NH_2_-truncated tau form(s) (Amadoro *et al.*, 2010, 2012; Corsetti *et al.*, 2015) has been recently recognized as one among the potentially targetable tau epitopes for promising Alzheimer’s disease immunotherapeutic interventions, being largely represented into CSF samples (Barthélemy *et al.*, 2016*a*, *b*; Sato *et al.*, 2018) and in autoptic specimens from affected subjects (Borreca *et al.*, 2018). Interestingly, previous *in vitro*, *ex vivo* and *in vivo* experiments from our research group have demonstrated that this short peptide when extracellularly administered to hippocampal neurons dynamically perturbs the plasma membranes—mainly of distal axonal compartments (Perini *et al.*, 2019)—by exerting a deleterious action on synaptic connectivity and plasticity being more likely internalized only after prolonged incubation times (Florenzano *et al.*, 2017; Borreca *et al.*, 2018). Moreover, studies have shown that tau antibodies can be readily taken up by neurons, promote the intracellular sequestration/clearance of pathological species by means of different mechanisms and prevent their release into the extracellular space followed by consequent spreading throughout the brain (Asuni *et al.*, 2007; Krishnamurthy *et al.*, 2011; Congdon *et al.*, 2013; Gu *et al.*, 2013; Collin *et al.*, 2014; Pedersen and Sigurdsson, 2015; Shamir *et al.*, 2016). After its i.v. administration in both healthy and disease mice, the 12A12mAb in circulation seems to be able to successfully penetrate the hippocampus and engage *in vivo* its target at a sufficient level to exert biologically relevant neuroprotective effects. The N‐terminal region of tau, despite the lack of the microtubule binding domains which abnormally aggregate to form paired helical filaments, is able to undergo higher order of oligomerization (Feinstein *et al.*, 2016) and, in this framework, the binding of 12A12mAb to the NH_2_htau may also prevent the trans-synaptic propagation of detrimental insoluble tau. Therefore, both extracellular and intracellular interaction between 12A12mAb and the NH_2_htau might be plausible routes by which immunization directed against this harmful, Alzheimer’s disease-relevant N-truncated tau specie(s) operates *in vivo*. Furthermore, although the immune system has been increasingly recognized as an important player in the immunotherapeutic approaches (Congdon and Sigurdsson, 2018; Katsinelos *et al.*, 2019), the finding that the cognitive skills improvement of 12A12mAb-injected Alzheimer’s disease Tg mice are paralleled by a strong and concomitant reduction of the disease-associated cerebral level of reactive gliosis further supports recent results showing that (i) antibody-mediated targeting of pathological tau *in vivo* does not necessarily required engagement of microglia that may *per se* induce deleterious neuroinflammation (Lee *et al.*, 2016); (ii) the neuroprotective mechanism action evoked by tau-based immunotherapy is more likely to rely on the direct neutralization of toxic extracellular species and/or on preventing their uptake by neurons (Congdon *et al.*, 2013; Gu *et al.*, 2013). In this regard, glial activation and neuroinflammation have been reported to severely impact on tau pathology directly, by participating to tau aggregation and degradation and spreading (Asai *et al.*, 2015; Yuan *et al.*, 2016; Bolos *et al.*, 2017; Hopp *et al.*, 2018), or indirectly, through a non-cell autonomous effect on neuronal signalling via cytokine and complement factor and gliotransmitter secretion (Liddelow *et al.*, 2017; Piacentini *et al.*, 2017; Litvinchuk *et al.*, 2018) and up-regulation of senescence-associated genes (Bussian *et al.*, 2018) and synapses pruning (Marttinen *et al.*, 2018; Vogels *et al.*, 2019).

Another challenging question is whether the neuroprotection offered by 12A12mAb can be further ameliorated *in vivo* following its prolonged administration, especially in more severe 3xTg animal model, or sustained even after its discontinuing immunization. Further investigations will be needed to better clarify the dose-dependent effect of 12A12mAb treatment on pathology and cognitive performance of Alzheimer’s disease Tg mice and how long the beneficial effect can last beyond the period of the immunization.

It is also worth stressing that—although mouse and human tau amino acidic sequences are similar—there are 14 amino acid differences in the N‐terminal region (Andorfer *et al.*, 2003; Bright *et al.*, 2015; Hernandez *et al.*, 2019). Nevertheless, the extreme N-terminal sequence of tau protein starting at D25 encompasses a not-canonical caspase(s) cleavage-site sequence (McStay *et al.*, 2008; Kumar *et al.*, 2014) which has been identified both in cellular (human SY5Y and rat PC12) and animal (Alzheimer’s disease11 mice) Alzheimer’s disease models (Rohn *et al.*, 2002; Corsetti *et al.*, 2008) and in human Alzheimer’s disease brains (Rohn *et al.*, 2002; Amadoro *et al.*, 2012; Quinn *et al.*, 2018). Moreover results from *in vitro* experiments and Tg animal models have shown that truncation plays a causative role in remodelling the highly flexible conformational ensemble of intrinsically disordered protein tau into Alzheimer’s disease-like pathological conformations (Novak *et al.*, 2018*b*). Conformational changes involving the amino-terminus of tau early occur in Alzheimer’s disease and other related tauopathies (Garcia-Sierra *et al.*, 2003). Consistently, Mukrasch *et al.*, have demonstrated that—although the largest part of tau441 amino acid sequence is devoid of any ordered structure—the N-terminal 50 residues of protein favour a compact conformation, as indicated by strong contacts within the residue stretch 1–20 and from this region to residues 30–50 (Mukrasch *et al.*, 2009). Therefore it is reasonable to hypothesize that, although the amino acid sequence of human and murine tau surrounding this epitope is divergent, 12A12mAb is more likely to recognize the newly generated, sequence- and structural-based immunoreactive determinants whose formation requires pathological truncation occurring under Alzheimer’s disease conditions at D25 residue both in human and rodent tau (Rohn *et al.*, 2002; Corsetti *et al.*, 2008; Quinn *et al.*, 2018; Amadoro *et al.*, 2019). In support of this finding and in line with the experimental evidence that temporary secondary structures occur in causal relation with tau neuropathology progression, both in isolated domains of the full-length protein and of some of its fragments (Mukrasch *et al.*, 2009; Avila *et al.*, 2016*b*; Fichou *et al.*, 2019), by means of molecular dynamics (MD) simulations and SAXS experiments, we have recently demonstrated that the short sequence including the 26–44 of N-terminal region of human tau—but not its reverse counterpart (tau44-26 peptide)—undergoes isolated β-bridges, α-helices and 3-helix which involve the Y29, T30, Q33, D34, Q35, E36 amino acid residues (Perini *et al.*, 2019). Importantly, these amino acid residues are present both in murine and primate tau sequence. Besides, the fact that 12A12mAb does not change the expression level of full-length tau but selectively reduces the endogenously produced 20–22 kDa tau fragment in both Alzheimer’s disease strains, as we showed in western blotting Figs 2 and 3, further strengthens the notion that a local conformational element (i.e. sequence- and structure-based immunoreactive epitope) is more likely to underlie its *in vivo* specificity in targeting the neo-epitope of the N-derived truncated pathological tau specie(s), both in human and mouse. Finally, since the epitopes cannot be predicted reliably from antigen primary amino acid sequences because some novel epitopes can arise exclusively due to the alteration of the molecule’s conformation (Opuni *et al.*, 2018), further experiments of immunoprecipitation followed by mass spectrometry and alanine epitope scanning mapping are needed to identify the crucial binding residues and the precise structure of N-terminal of tau protein that are directly involved in the interaction with 12A12mAb.

Concerning the interplay occurring *in vivo* between APP/Aβ and tau pathologies, according to the classical Aβ cascade hypothesis aberrant changes of tau metabolism are considered downstream of Aβ pathology which acts as initial trigger (Hardy and Selkoe, 2002; De Strooper and Karran, 2016). Consistently, compelling studies have demonstrated that Aβ can potentiate tau abnormalities (Gotz *et al.*, 2001; Lewis *et al.*, 2001; Oddo *et al.*, 2004; Bolmont *et al.*, 2007) and that an enhanced neuropathology occurs following *in vivo* interaction between Aβ and tau (Gotz *et al.*, 2001; Lewis *et al.*, 2001; Hurtado *et al.*, 2010; Bennett *et al.*, 2017; Pontecorvo *et al.*, 2017; He *et al.*, 2018; Jacobs *et al.*, 2018; Quiroz *et al.*, 2018). In this regard, our findings highlighting a novel mechanistic interplay between APP/Aβ and tau at synapses fit more well with other studies showing that changes in tau metabolism precede Aβ pathology in aged and Alzheimer’s disease brains (Braak and Del Tredici, 2004; Schonheit *et al.*, 2004; Braak *et al.*, 2013; Jack *et al.*, 2013) and that the removal of pathogenetic species of tau can prevent *in vivo* the deleterious effect of both Aβ and tau (Oddo *et al.*, 2006; Castillo-Carranza *et al.*, 2015; Dai *et al.*, 2017, 2018; Rajamohamedsait *et al.*, 2017). Remarkably, the spreading/propagation of tau neuropathology into the Aβ plaque-bearing cerebral cortex is associated with the transition from the preclinical (asymptomatic) to the clinical (symptomatic) stage of Alzheimer’s disease (Delacourte *et al.*, 1999; Wang *et al.*, 2016; Pontecorvo *et al.*, 2017). Furthermore, although the tau pathology to evolve to full-blown Alzheimer’s disease requires the concomitant presence of Aβ pathology (Braak and Del Tredici, 2011; Duyckaerts, 2011; Jack *et al.*, 2013; Crary *et al.*, 2014), the failure of anti-Aβ therapies in preventing the disease progression suggests that Alzheimer’s disease pathogenesis might be driven by tau independently of Aβ (Giacobini and Gold, 2013). However, whether Aβ is necessary for tau neurotoxicity or whether the reverse is true is still an open question (Ashe and Zahs, 2010). On the other hand, recent data also suggest that tau and Aβ may be independent processes and reciprocally interact over the evolution of Alzheimer’s disease (Small and Duff, 2008; Mondragón-Rodríguez *et al.*, 2010). In this context, co-occurrence between tau and Aβ within neuronal processes and synaptic compartments has been described in Alzheimer’s disease (Hoover *et al.*, 2010; Ittner *et al.*, 2010; Zempel *et al.*, 2010; Amadoro *et al.*, 2012; Miller *et al.*, 2014) and synaptic abnormalities occur in aging Tg2576 and 3xTg mice (Spires-Jones *et al.*, 2007; Nisticò *et al.*, 2012; Ameen-Ali *et al.*, 2017). Aβ and tau pathologies exert synergistic effects on neuronal morphology/function (Rhein *et al.*, 2009) particularly at synapses (Hoover *et al.*, 2010; Ittner *et al.*, 2010; Takahashi *et al.*, 2010; Amadoro *et al.*, 2012) believed to initiate Alzheimer’s disease progression (Selkoe, 2002), indicating that passive immunization with 12A12mAb can contribute to improve disease-associated mnestic disabilities at its early phases by preventing both pathognomonic toxic proteins from damaging synaptic connectivity in pathologically relevant vulnerable neuronal circuits. Furthermore, a recent hypothesis also suggests that synaptic dysfunction in Alzheimer’s disease is triggered by impairment of APP metabolism which further progresses via tau pathology (Gulisano *et al.*, 2018; Kametani and Hasegawa, 2018; Schreurs *et al.*, 2018). Consistently, an increased level of APP and/or its C-terminal fragments are able to induce axonal and synaptic defects (Rusu *et al.*, 2007; Rodrigues *et al.*, 2012; Deyts *et al.*, 2016; Xu *et al.*, 2016) associated with mis-localization of tau (Blurton-Jones and Laferla, 2006; Hochgrafe *et al.*, 2013). Overexpression of APP promotes *per se* the seeded aggregation of intracellular tau in cultured cell, suggesting that APP, rather than Aβ, can work as a receptor of abnormal tau fibrils (Takahashi *et al.*, 2015) by accelerating *in vivo* internalization in neurons (Holmes *et al.*, 2013; Mirbaha *et al.*, 2015) followed by pathological accumulation and propagation. Besides, both soluble/prefibrillar extracellular toxic Aβ and tau can damage the synaptic terminals in APP-dependent manner (Puzzo *et al.*, 2017; Wang *et al.*, 2017), suggesting a translation potential of 12A12mAb for APP-targeted therapy in patients.

Concerning the routes by which the 12A12mAb-mediated removal of the NH_2_htau can affect the cross-talk between Aβ and tau neuropathology or interfere upstream with APP metabolism and/or its processing *in vivo*, both cell- and non-cell autonomous action mechanisms (Alasmari *et al.*, 2018) should be taken into account by operating in alternative but not mutually exclusive manners and by acting at different transcriptional (Bright *et al.*, 2015; Zhang *et al.*, 2018), translation (Asuni *et al.*, 2014; Borreca *et al.*, 2016; Meier *et al.*, 2016; Koren *et al.*, 2019) and post-translational (Amadoro *et al.*, 2012) levels. Furthermore, variations in the type of mechanism(s) engaged *in vivo* by 12A12mAb in two APP mouse models analysed and/or dissimilarity in their temporal progression of plaque deposition (Ameen-Ali *et al.*, 2017) are more likely to account for the difference in the magnitude of antibody effect(s) on APP/Aβ mis-processing. At the present, *in vitro*, *ex vivo* and *in vivo* experiments are being performed by our research group to better clarify this important issue.

In conclusion, the present investigation not only highlights a novel dynamic positive feed-forward regulation between APP/Aβ and N-truncated tau *in vivo* by reinforcing the concept of pathological tau as main therapeutic target of Alzheimer’s disease but also hopefully helps to design more efficacious and safety tau-directed interventions by prospecting the 12A12mAb as beneficial and disease-modifying approach for the cure of Alzheimer’s disease and other tauopathies.

## Supplementary material

Supplementary material is available at *Brain Communications* online.

## Supplementary Material

fcaa039_Supplementary_Data

## Abbreviations

Aβ =: amyloid-β peptides
APP =: amyloid precursor protein
DO =: displaced object
EE =: energy expenditure
ELISA =: enzyme-linked immunosorbent assay
FO =: familiar one
i.v =: intravenous
LTP =: long-term potentiation
mAB =: monoclonal antibody
NO =: novel object
NOR =: novel object recognition
PBS =: phosphate-buffered-saline
PBST =: PBS containing 0.05% Tween-20
PI =: preference index
RT =: room temperature
SO =: stationary object
TBS =: tris-buffered saline
Tg =: transgenic
WT =: wild-type
