# Supplementary material for: Passive immunotherapy for N-truncated tau ameliorates the cognitive deficits in two mouse Alzheimer’s disease models
Source: Brain Commun. 2020 Apr 6;2(1):fcaa039. doi: 10.1093/braincomms/fcaa039 (PMC7425324; doi:10.1093/braincomms/fcaa039)
Supplement: fcaa039_Supplementary_Data [file fcaa039_supplementary_data.pdf]

Suppl. Figure 1

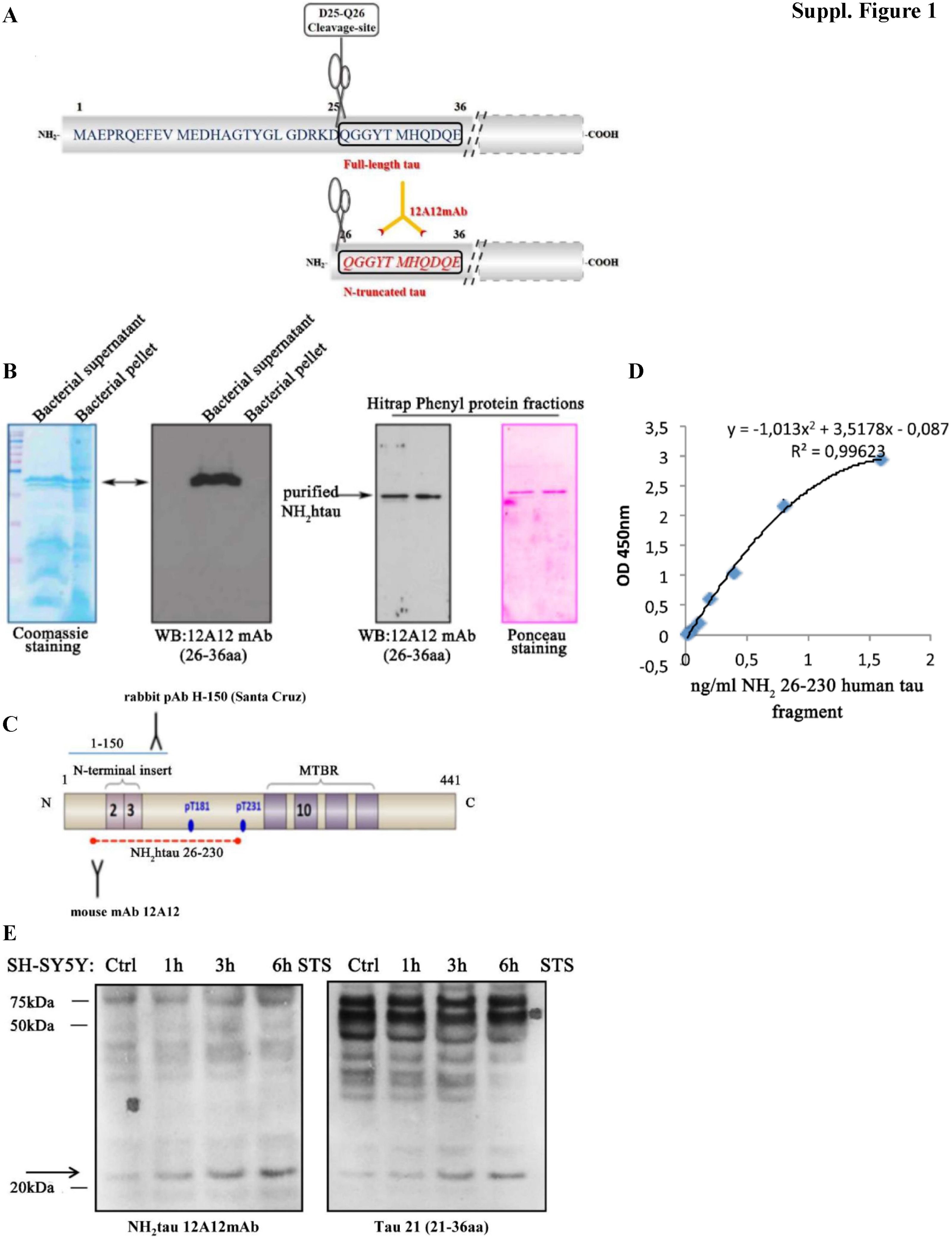

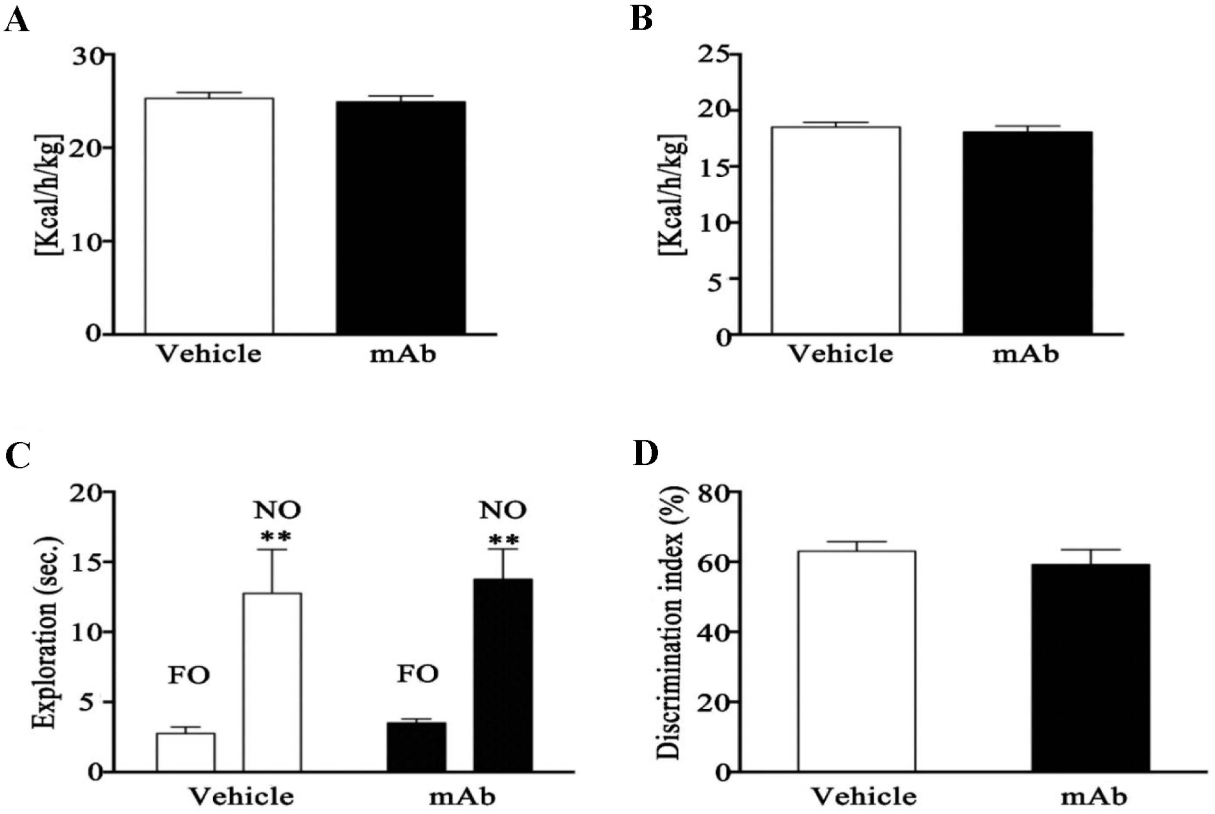
A
30
20
10
0
[Kcal/h/kg]
Vehicle
mAb
B
25
20
15
10
5
0
[Kcal/h/kg]
Vehicle
mAb
C
20
15
10
5
0
Exploration (sec.)
FO
NO
**
FO
NO
**
Vehicle
mAb
D
80
60
40
20
0
Discrimination index (%)
Vehicle
mAb

Suppl. Figure 3

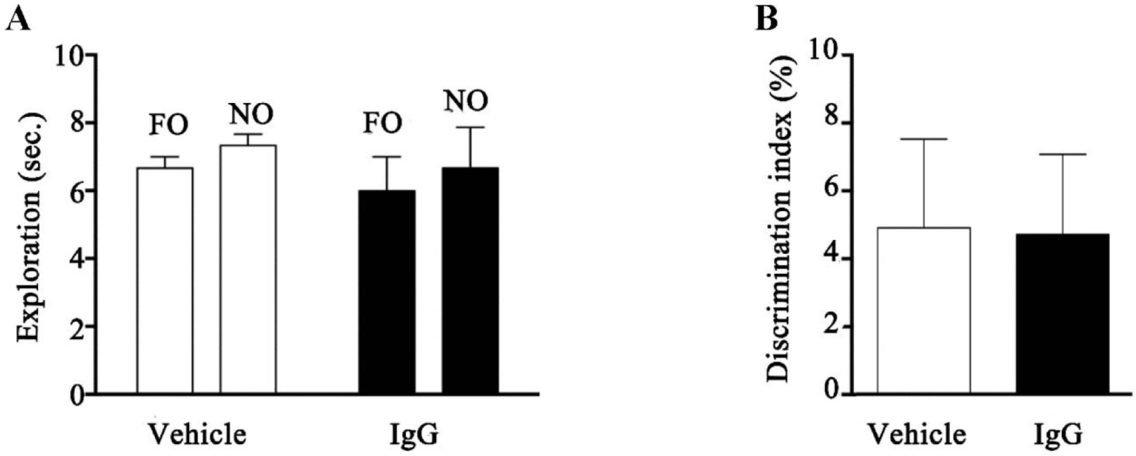

Suppl. Figure 4

A

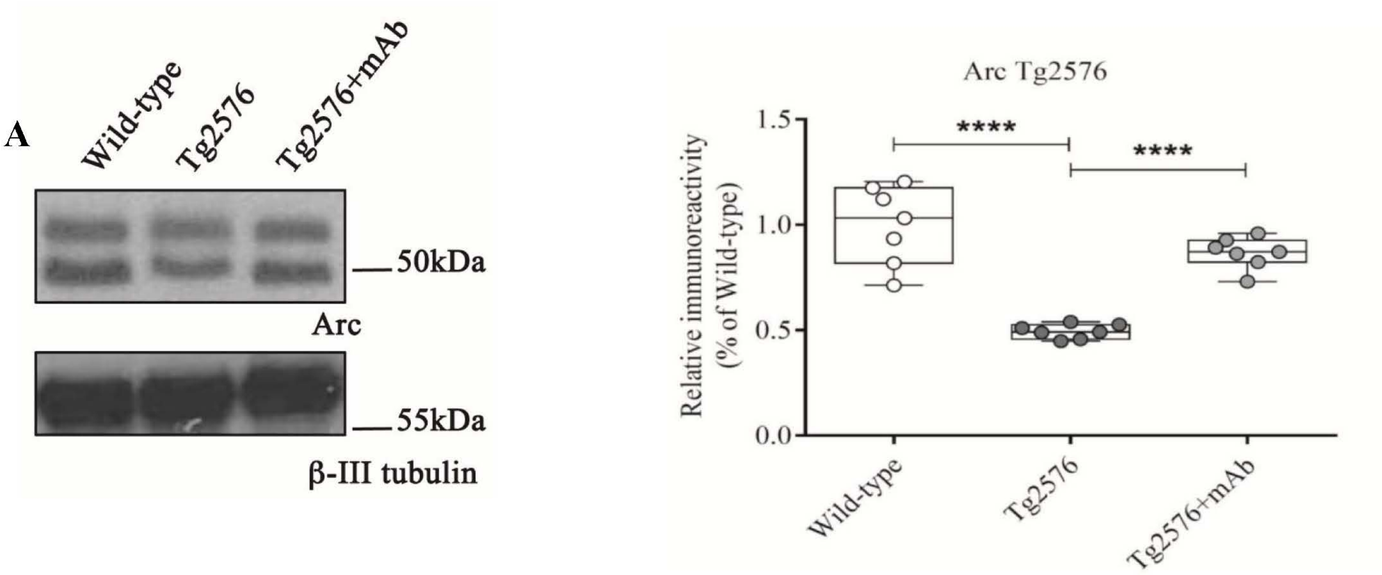

B

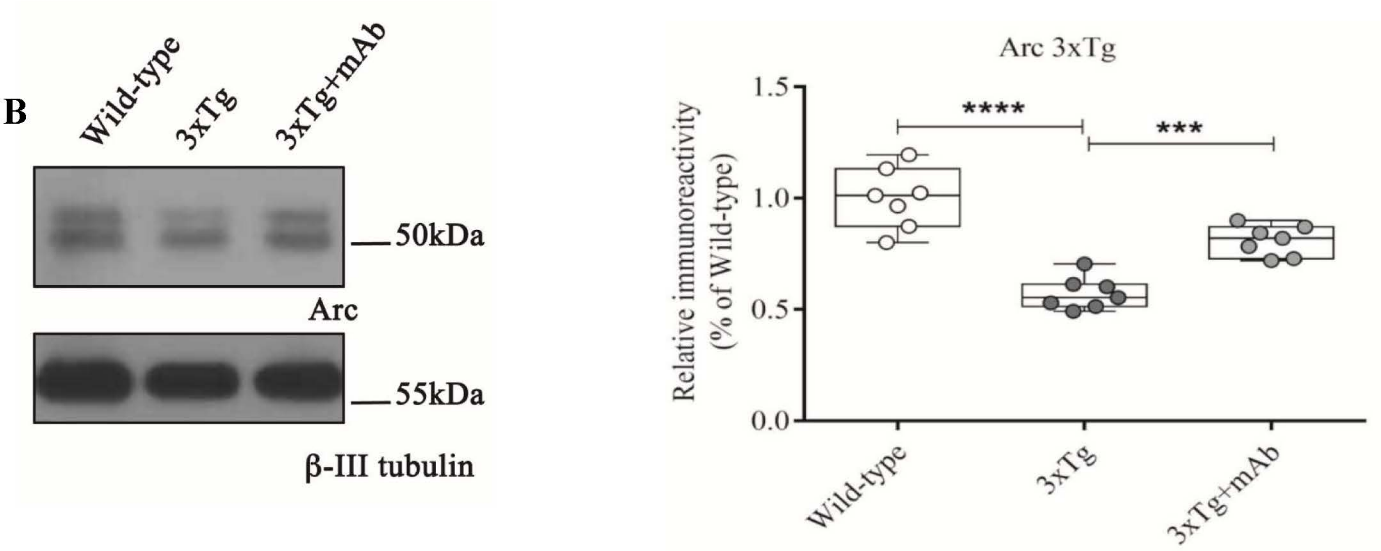

Suppl. Fig. 5

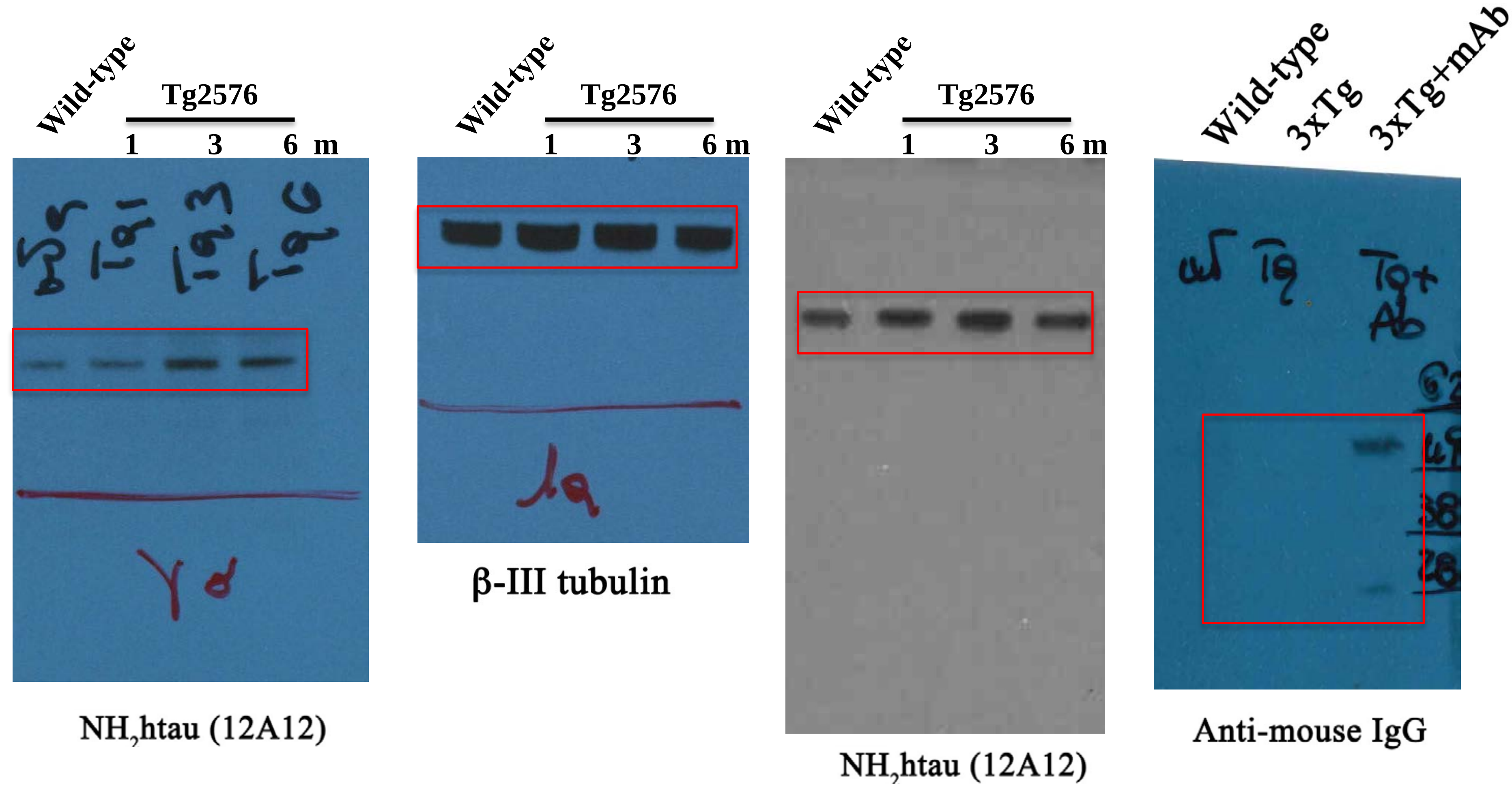

Fig1. A | Fig. 1A | Fig.1B | Fig. 1C

Suppl. Fig. 6

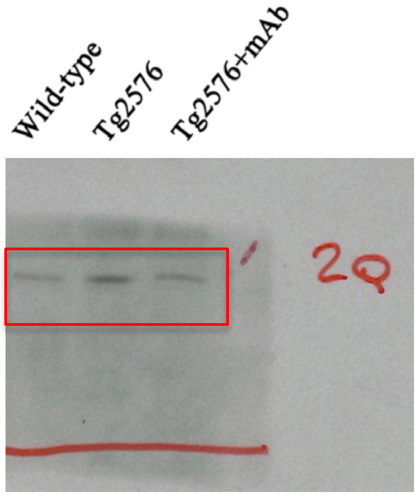

NH$_2$htau (12A12)

Fig. 2A

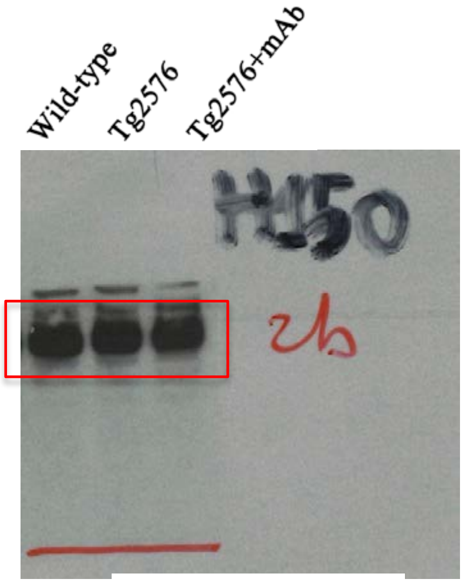

Total tau (H150)

Fig. 2B

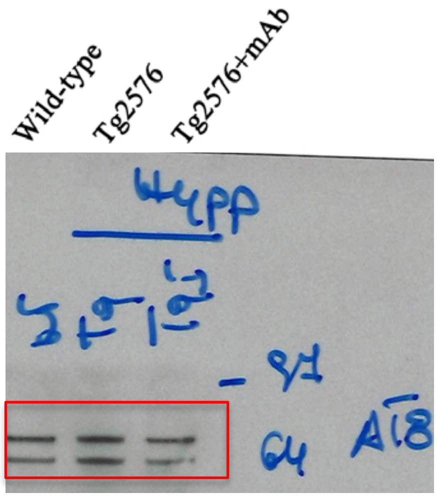

APP (22C11)

Fig. 2C

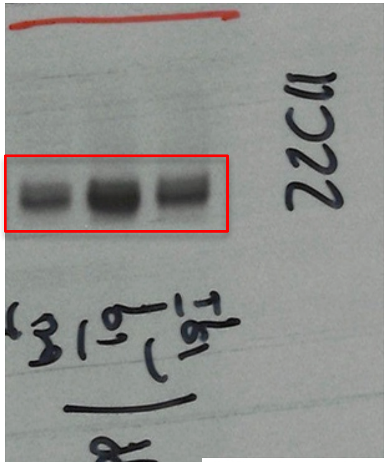

Aβ(6E10)

Fig. 2D

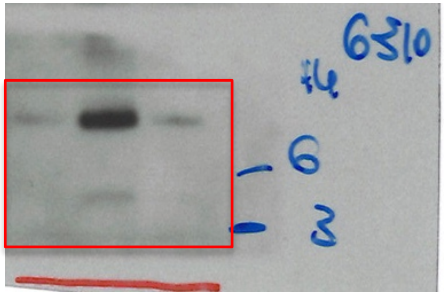

Aβ(6E10)

Fig. 2E

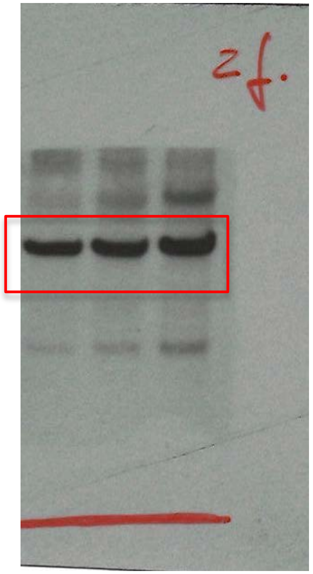

β-III tubulin

Fig. 2F

Suppl. Fig.7

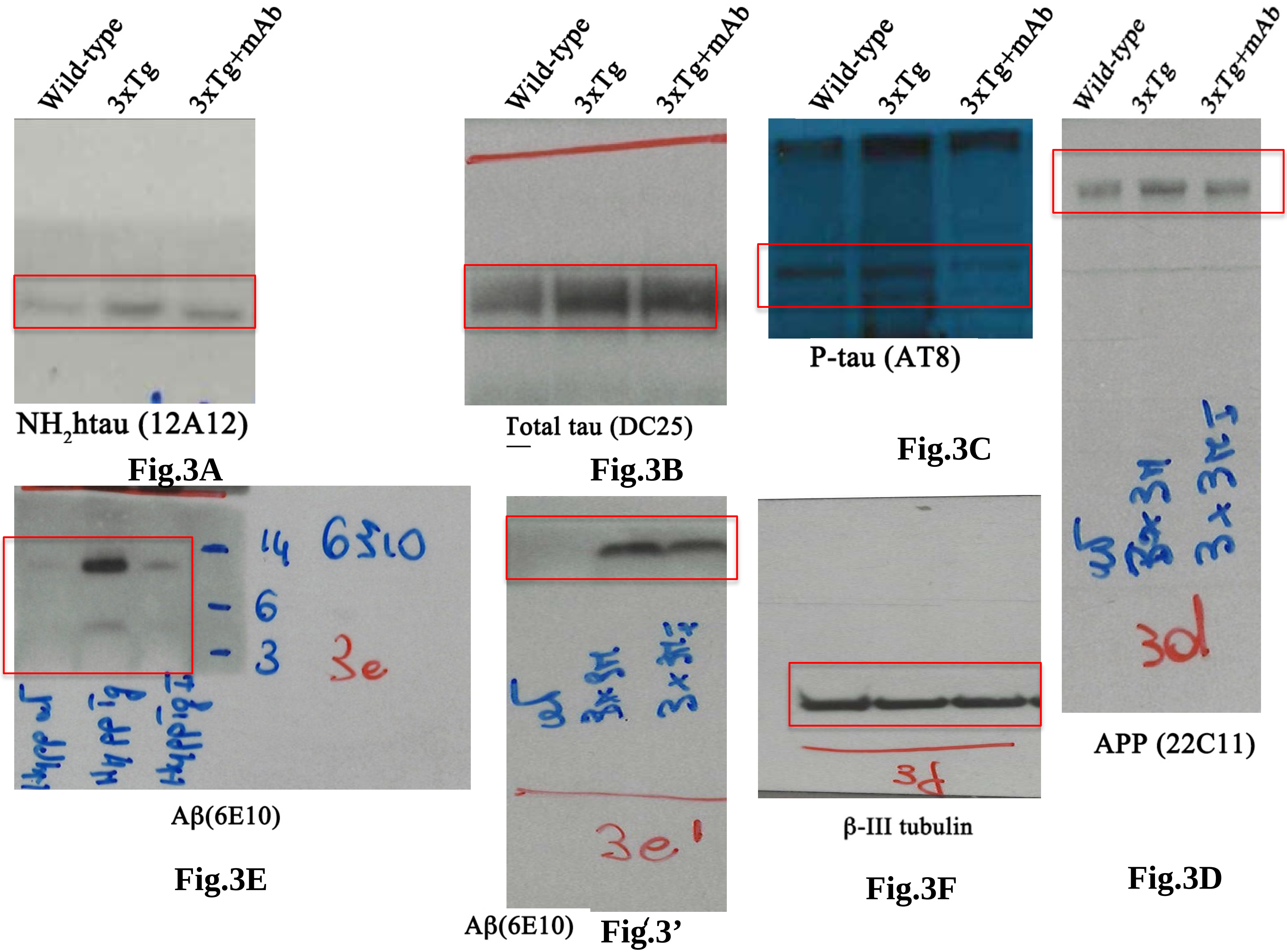

Wild-type
3xTg
3xTg+mAb
NH$_2$htau (12A12)
Fig.3A
Wild-type
3xTg
3xTg+mAb
Total tau (DC25)
Fig.3B
Wild-type
3xTg
3xTg+mAb
P-tau (AT8)
Fig.3C
Wild-type
3xTg
3xTg+mAb
APP (22C11)
Fig.3D
Aβ(6E10)
Fig.3E
Aβ(6E10)
Fig.3'
β-III tubulin
Fig.3F

Suppl. Fig. 8

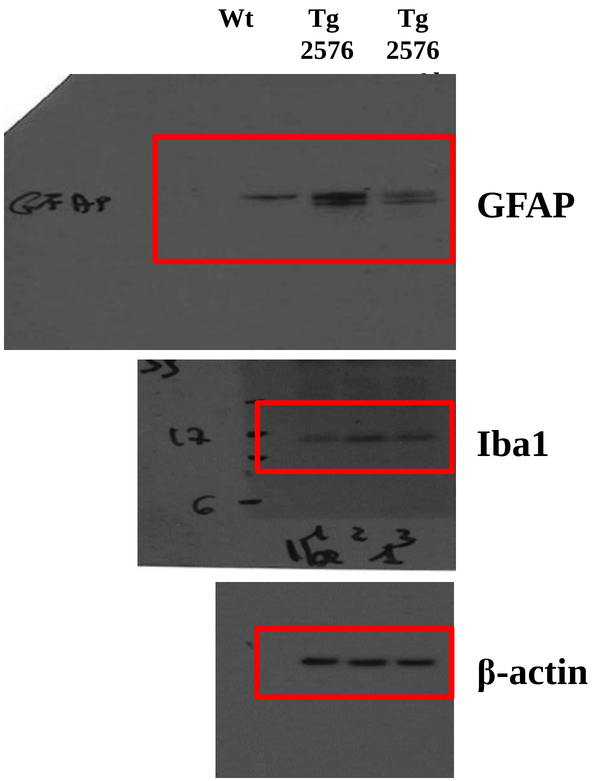

Fig. 8 A

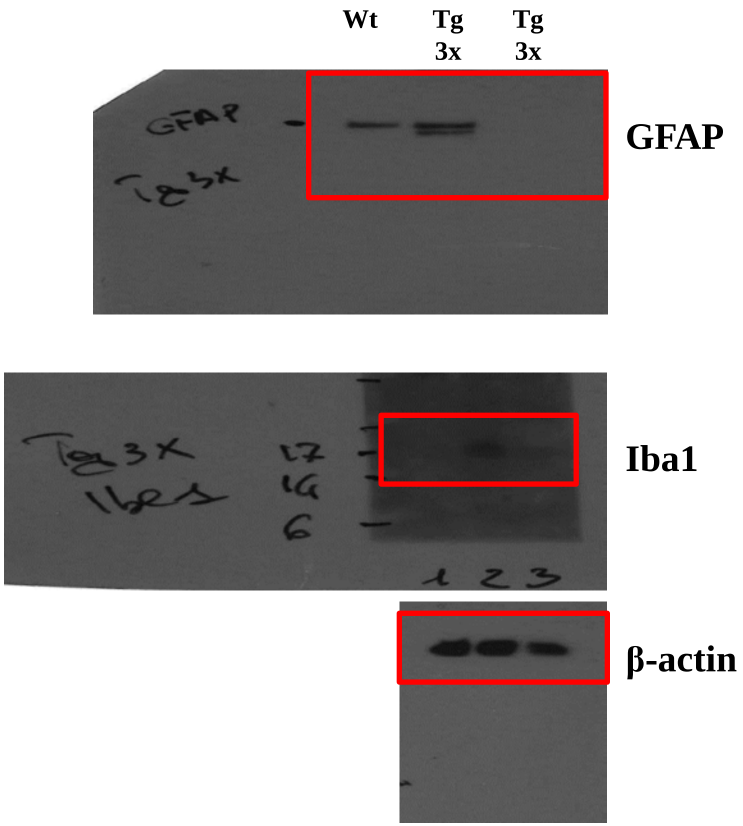

Fig. 8 B

Suppl. Fig. 9

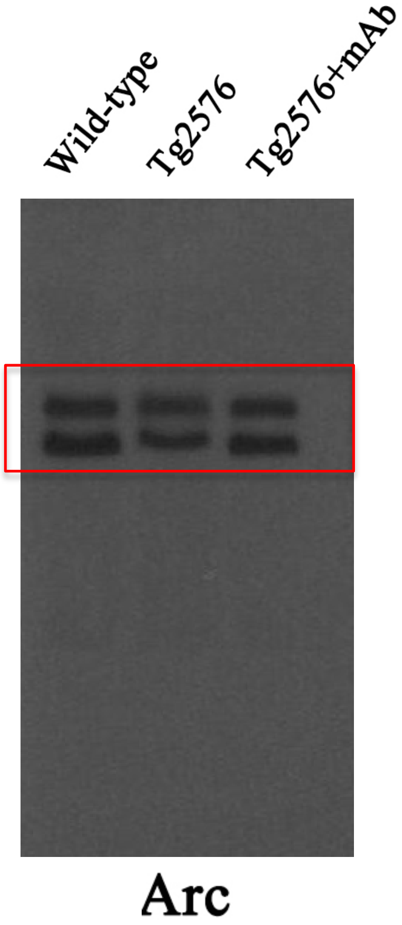

Suppl Fig. 4A

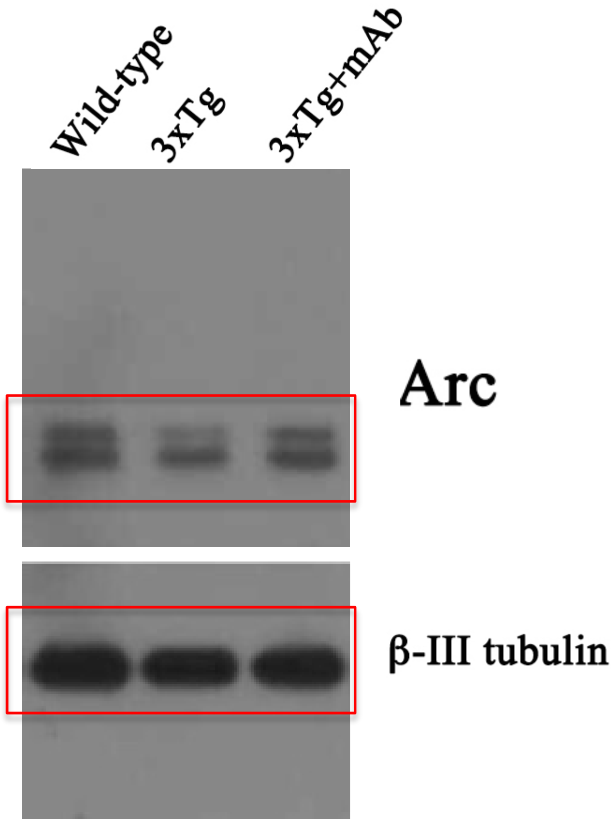

Suppl Fig. 4B

Supplementary figures legend

Figure S1. 12A12mAb binds the recombinant, purified $NH_2$26-230 tau fragment and the endogenously-produced, native 20-22kDa tau fragment in differentiated human SH-SY5Y undergoing apoptosis following STS treatment.

A) Schematic of 12A12mAb specificity towards the newly-created Δ-25NH_2tau(Q26-36aa)-terminus of degradation product(s) at N-terminal domain of tau. Letters refer to the single aminoacid code while the number refers to the position along the length of the full-length human tau441 isoform.

B-C-D) SDS-PAGE analysis probing with 12A12mAb was carried out to check its ability of binding the recombinant, purified $NH_2$26-230 tau fragment (B). The $NH_2$26-230 fragment calibration curve (D) was calculated by sandwich Enzyme-linked immunosorbent assay (ELISA) 12A12/H150 (C) by means of:

-Capture antibody: mouse 12A12 (26-36aa human tau protein)

-Detecting antibody: rabbit H150 Santa Cruz sc-5587 (100-150aa human tau protein).

E) Western blotting analysis was carried out on equal amounts of total protein extract (30μg) from human differentiated SH-SY5Y neuroblastoma cell line exposed to 1 μM staurosporine (STS) for different period of times (1, 3, 6 hrs). Immunoblots were probed with 12A12mAb and with commercial Tau 21 (21-36aa) (pAb AHB0371 Biosource International) detecting all the 6 isoforms of protein. 12A12mAb (formerly Caspase-Cleaved protein-$NH_2$4268 tau antiserum, Amadoro et al., 2012) is neoepitope antibody directed against the N-terminal sequence of human tau protein $DRKD_{(25)}$QGGYTMHQDQE which encompasses a conserved caspase(s)-cleavage site (Rohn et al., 2002; Canu et al., 1998). This antibody recognizes the newly-created Δ-25 NH_2tau(Q26-36aa)-terminus of degradation product(s) at N-end of both murine and human tau without cross-reaction with the same aminoacidic stretch from full-length, intact isoforms of protein (Amadoro et al., 2019). For normalization of the samples' loading, β-actin was used. Arrows on the right side indicate the Molecular Weight (kDa) of bands calculated from migration of standard proteins. Notice that mAb specifically detected band corresponding to the 20-22kDa NH_2-truncated tau fragment but did not recognize the full-length tau isoforms shown by immunoreactivity with commercial Tau 21 antiserum.

Figure S2. 12A12mAb administration does not change the behaviour and the energetic metabolism in wild-type, healthy, mice.

A-B) Bar graphs report the mean of 48hrs Energy expenditure (EE) as heat (kcal/h per kg of body weight) and the resting EE (REE) emitted/produced by vehicle- and mAb-treated wild-type mice under standard feeding conditions in 48hrs continuous indirect calorimetry analysis, respectively. Data were expressed as means(±SEM) followed by unpaired sample t-test.

C-D) Bar graphs report the time of exploration (sec) of familiar object (FO) in comparison to novel object (NO) (C) and the discrimination index in vehicle- and mAb-treated wild-type healthy mice assessed for cognitive performance in novel object recognition (NOR) task. Discrimination index (DI) between objects was expressed as the difference in time spent exploring the novel object (NO) and the familiar object (FO) divided by the total time exploring both objects (DI=(N0–F0)/(N0+F0)x100%). Data were expressed as means(±SEM) followed by two-way ANOVA Tukey's post-hoc test and unpaired t-test, respectively.

Figure S3. Not-specific, control IgG administration does not improve the deficits of recognition memory in cognitively-impaired Tg-AD mice.

A) Time of exploration (sec) of familiar object (FO) and novel object (NO) in vehicle- and IgG-treated 6-month-old Tg2576 mice assessed for performance in NOR task. Two-way ANOVA showed no statistically significant change in the animals' exploratory activity of NO in comparison with FO (B) Discrimination Index (DI) of vehicle- and IgG-treated Tg2576 mice assessed for performance in NOR task. Discrimination index (DI) between objects was expressed as the difference in time spent exploring the novel object (NO) and the familiar object (FO) divided by the total time exploring both objects (DI=(NO–FO)/(NO+FO)x100%). Unpaired sample t-test of data showed no statistically significant effect of both treatments towards the transgenic animals' deficits in discriminating the NO.

Figure S4. The activity-regulated cytoskeleton-associated protein Arc is upregulated in synapses from 12A12mAb-vaccinated Tg-AD mice of both genetic backgrounds.

A-B) Representative blots (n=4) of SDS-PAGE Western blotting analysis (left) on isolated synaptosomal preparations from hippocampal region of animals from three experimental groups (wild-type, Tg-AD and Tg-AD+mAb) of both strains (Tg2576, 3xTg) sacrificed at the end of test session to assess the content of the activity-regulated cytoskeleton-associated protein Arc which is

normally evoked by short-term memory/learning task. β-III tubulin was used as loading control and relative densitometric quantifications are reported (right). Arrows on the right side indicate the molecular weight (kDa) of bands calculated from migration of standard proteins. Full uncropped blots are available in Suppl. Fig. 9. Plots show pooled data and statistically significant differences (see details in the main text) were calculated by ANOVA followed by post-hoc test for multiple comparison among more than two groups. $p<0.05$ was accepted as statistically significant (*$p<0.05$; **$p<0.01$; ***$p<0.0005$; ****$p<0.0001$).

Figure S5-9

Full, non-cropped images are shown. Numbers and letters refer to the cropped blots of Western Blotting experiments reported in Fig.1, 2,3,8,S4.
